# Enrichment of H3K9me2 on Unsynapsed Chromatin in *Caenorhabditis elegans* Does Not Target *de Novo* Sites

**DOI:** 10.1534/g3.115.019828

**Published:** 2015-07-08

**Authors:** Yiqing Guo, Bing Yang, Yini Li, Xia Xu, Eleanor M. Maine

**Affiliations:** Department of Biology, Syracuse University, Syracuse, New York 13244

**Keywords:** meiosis, germ cells, gene regulation, histone modification, MET-2

## Abstract

Many organisms alter the chromatin state of unsynapsed chromosomes during meiotic prophase, a phenomenon hypothesized to function in maintaining germline integrity. In *Caenorhabditis elegans*, histone H3 lysine 9 dimethylation (H3K9me2) is detected by immunolabeling as enriched on unsynapsed meiotic chromosomes. Loss of the SET domain protein, MET-2, greatly reduces H3K9me2 abundance and results in germline mortality. Here, we used *him-8* mutations to disable X chromosome synapsis and performed a combination of molecular assays to map the sites of H3K9me2 accumulation, evaluate H3K9me2 abundance in germline *vs.* whole animals, and evaluate the impact of H3K9me2 loss on the germline transcriptome. Our data indicate that H3K9me2 is elevated broadly across the X chromosome and at defined X chromosomal sites in *him-8* adults compared with controls. H3K9me2 levels are also elevated to a lesser degree at sites on synapsed chromosomes in *him-8* adults compared with controls. These results suggest that MET-2 activity is elevated in *him-8* mutants generally as well as targeted preferentially to the unsynapsed X. Abundance of H3K9me2 and other histone H3 modifications is low in germline chromatin compared with whole animals, which may facilitate genome reprogramming during gametogenesis. Loss of H3K9me2 has a subtle impact on the *him-8* germline transcriptome, suggesting H3K9me2 may not be a major regulator of developmental gene expression in *C. elegans*. We hypothesize H3K9me2 may have a structural function critical for germline immortality, and a greater abundance of these marks may be required when a chromosome does not synapse.

In the germ line, as in all developing tissues, gene expression and chromosome segregation are in part regulated by chromatin structure. Moreover, as the tissue that gives rise to subsequent generations, the germ line undergoes distinctive processes during gamete formation. One distinctive aspect of germline chromatin regulation conserved in many animal species is a process called meiotic sex chromosome inactivation (MSCI), whereby the heterogametic sex chromosomes acquire histone modifications during first meiotic prophase that are consistent with a heterochromatic state (reviewed by Handel 2004; Kelly and Aramayo 2007; Maine 2010; Turner 2007). Similarly during first meiotic prophase, any homologous chromosomes that fail to synapse because of mutation, translocation, or duplication acquire elevated levels of heterochromatin-associated histone modifications in a process variably termed meiotic silencing of unpaired chromatin or meiotic silencing of unsynapsed chromosomes (MSUC) (Maine 2010) . These meiosis-specific chromatin changes, observed in many animal species (including mammals, birds, nematodes, some insects), have been implicated in transcriptional and checkpoint control and have been hypothesized to play additional roles in regulating genome stability.

In *C. elegans* (X0) males, the X chromosome acquires a relatively high level of histone H3 lysine 9 dimethylation (H3K9me2) during first meiotic prophase (Kelly *et al.* 2002; Bean *et al.* 2004). H3K9me2 levels also increase during early meiosis on unsynapsed homologs present in certain mutant backgrounds, *e.g.*, *him-8* mutant hermaphrodites where the X chromosomes fail to synapse (because HIM-8 mediates X chromosome pairing), or on single-copy free chromosomal duplications, *e.g.*, *sDp3* (Bean *et al.* 2004). MET-2 (the SETDB1 ortholog, histone methyltransferase-2) is primarily responsible for H3K9me2 marks in the *C. elegans* germ line, as H3K9me2 is not detectable by indirect immunofluorescence labeling of whole gonads isolated from *met-2(0)* mutants (Bessler *et al.* 2010). *In situ* hybridization analysis of X-linked oogenic genes, normally expressed in late pachytene, revealed a sharp reduction in mRNA levels in XO hermaphrodites (Bean *et al.* 2004; Jaramillo-Lambert and Engebrecht 2010), suggesting the elevated H3K9me2 may limit transcription of X-linked genes.

We previously showed that meiotic H3K9me2 distribution is severely altered when certain components of the germline small RNA network are mutated (Maine *et al.* 2005; She *et al.* 2009). Specifically, H3K9me2 enrichment on unsynapsed chromosomes and chromosomal regions is reduced when components of the CSR-1 (chromosome-segregation and RNAi deficient) Argonaute pathway are absent, including EGO-1 RNA-directed RNA polymerase (RdRP); DRH-3 helicase; the Tudor domain protein, EKL-1; and CSR-1. Unsynapsed chromosomes evaluated for H3K9me2 enrichment included the male X, hermaphrodite X chromosomes in *him-8* mutants, the large free duplication *sDp3*, and several extrachromosomal arrays (Maine *et al.* 2005; She *et al.* 2009). In addition, ectopic H3K9me2 is detected on synapsed chromosomes in the absence of CSR-1, leading us to propose that CSR-1 activity might limit deposition of H3K9me2 marks at synapsed regions (She *et al.* 2009). This phenotype is consistent with the proposed role for CSR-1 as a positive regulator of germline gene expression (Claycomb *et al.* 2009; Seth *et al.* 2013; Wedeles *et al.* 2013).

To better understand the biological function of H3K9me2 enrichment as well as the mechanism by which these marks are targeted to particular genomic sites, we sought to map the sites of H3K9me2 enrichment on synapsed and unsynapsed chromosomes. We used chromatin immunoprecipitation followed by sequencing of precipitated DNA (chromatin immunoprecipitation sequencing; ChIP-seq) to evaluate the H3K9me2 pattern in control adult hermaphrodites and adult *him-8* mutants where the X chromosomes fail to synapse during meiosis. The existing *C. elegans* literature regarding H3K9me2 distribution in the meiotic germ line is based on indirect immunofluorescence assays. The use of ChIP-seq allowed us to obtain a detailed picture of H3K9me2 distribution across the genome. We detect a similar pattern of H3K9me2 distribution in control and *him-8* hermaphrodites, with a higher proportion of H3K9me2-associated sequence reads derived from the X chromosome in *him-8* hermaphrodites compared with controls. In addition, H3K9me2 levels are higher in *him-8* at many sites dispersed across the genome. Protein blotting similarly detects an increased H3K9me2 abundance in *him-8* relative to controls. Taken together, these data indicate that H3K9me2 levels are generally elevated in the *him-8* genome, with an especial enrichment on the X. Messenger RNA-seq analysis reveals that loss of H3K9me2 has a subtle effect on the gonad transcriptome. Interestingly in light of the link to RNA mechanisms, H3K9me2 levels anti-correlate with candidate targets of CSR-1/Argonaute activity and strongly correlate with candidate targets of WAGO-1 (worm Argonaute protein)/Argonaute activity.

## Materials and Methods

### Strains

*C. elegans* strains were cultured using standard methods (Brenner 1974). *C. elegans* var. Bristol (N2) is the wild-type parent strain for all mutations used in this study. Information on specific genes, alleles, and nomenclature can be found at www.wormbase.org. Strains used for H3K9me2 ChIP-seq analysis were *fer-1(b232ts);him-8(e1489)* and *fer-1(b232ts)*. Strains used for gonad mRNA-seq were *met-2(n4256);him-8(tm611)* and *him-8(tm611)*. Strains carrying multiple mutations were generated by standard methods and confirmed by polymerase chain reaction (PCR) and/or sequencing. The strain deleted for all 12 *wago* clade genes (MAGO12) (Gu *et al.* 2009) was a gift from Darryl Conte. To obtain MAGO12 males for indirect immunofluorescence, L4 hermaphrodites were placed at 30° for 4 hr, males were collected from among their progeny and used to set up matings, and males from subsequent generations were immunolabeled.

The two *him-8* alleles used in our strains disrupt production of functional HIM-8 protein and, consequently, prevent X chromosome synapsis and pairing (Phillips *et al.* 2005). We performed indirect immunofluorescence to confirm that H3K9me2 accumulated on the unsynapsed X chromosomes in each *him-8* strain and failed to accumulate in each *met*-2 strain, as expected (Supporting Information, Figure S1). We used *him-8(tm611)* for mRNA-seq because it contains a small deletion that makes an excellent internal control for the sequencing data.

### Indirect immunofluorescence and Western blotting

H3K9me2 indirect immunofluorescence was performed as described (She *et al.* 2009). Briefly, gonads were dissected in 0.1−0.2 mM levamisole/phosphate-buffered saline (PBS) in a deep-well slide and fixed by adding 200 μL of a 3% PFA/1× PBS solution to the well and incubating at room temperature (RT) for 5 min. Tissue was washed 3× in PBST (1× PBS/0.1% Tween-20), blocked in 30% goat serum/PBST for 1−2 hr, and incubated with anti-H3K9me2 (1:200 dilution, Abcam 1220) at 15° overnight. Tissue was washed 3× 15 min in PBST, incubated for 2 hr with Alexa488-conjugated goat antimouse secondary antibody (1:200 dilution, Invitrogen) at RT, and washed 3× in PBS; DAPI was included in the penultimate wash. Tissue was mounted on slides in VECTASHIELD medium (Vector Labs). Images were captured on a Zeiss Axioscope.

Western blots were prepared using standard protocols. Gonads were dissected from hermaphrodites at L4 stage + ∼18−24 hr. Animals were washed 2X in M9 + protease inhibitor cocktail (Roche) and dissected in fresh M9 + protease inhibitor. Dissected gonads were transferred to a well with fresh solution; when ∼150 gonads were accumulated, material was centrifuged, excess solution removed, and tissue was frozen on dry ice. For whole worms, the specified number of animals was washed in M9, transferred to a tube, spun down, excess solution removed, and frozen on dry ice. To prepare samples, an appropriate amount of 4× loading buffer was added, and sample was boiled for 10 min, cooled down on ice, and centrifuged. Supernatant was transferred to a new tube and volume was measured. Total volume was adjusted by addition of 1× loading buffer, and appropriate amounts were loaded onto gels for histone detection. Typically, the adjusted volume was 32 μL; 16 μL was used for testing histone H3 modification, and 8 μL was used for testing histone H3. Pierce SuperSignal West Pico (#34080) or Femto (#34094) detection substrate was used to visualize protein. We noted that the Femto substrate consistently detected rarer modifications, H3K9me2 and H3K4me3, at a higher level relative to total H3 than did the Pico substrate. In contrast, we did not observe this difference when assaying protein that is present at a relatively greater level, *e.g.*, gonad H3K4me1 was detected at 80% of whole animal H3K4me1 by both the Pico and Femto substrates. We hypothesize that detection at the lower limit of the Pico substrate sensitivity is unreliable, and included only Femto detection data for H3K9me2 and H3K4me3 in Figure 6. Quantification was performed using Image J software (Gassmann *et al.* 2009).

### ChIP-seq

To obtain adults for ChIP, we collected embryos from animals grown at 15°, hatched them into M9 medium, collected the synchronized L1s, raised them in liquid medium at 22° for ∼60 hr, and harvested adults. For *him-8* strains, hermaphrodites were separated from males using 35 micrometer nylon mesh (Sefar). Animals were fixed in 1% formaldehyde for 10−20 min at 22° (RT), frozen in liquid nitrogen, and stored at −80°. Lysis buffer (50 mM Tris-HCl, 1% SDS, 10 mM ethylenediaminetetraacetic acid (EDTA), 1× protease inhibitor cocktail; Roche 04693132001) was added to frozen samples, which were then thawed on ice. Tissue was disrupted and chromatin was sheared with a probe sonicator (Sonic Dismembrator, Fisher Scientific) 8 × 10 sec on setting 7 (output power ∼24 Watts), with cooling on ice for 2 min after each pulse. Protein concentration was measured by Bradford assay. A total of 1−2 mg of total protein extract (100 μL) was added to 900 μL of dilution buffer (1.1% Triton X-100, 1.2 mM EDTA, 16.7 mM Tris-HCl, 167 mM NaCl, 1× protease inhibitor cocktail, Roche), and precleared by addition of DynaBeads. Anti-H3K9me2 (Abcam1220) was used to detected histone modification. Normal mouse IgG (Invitrogen; 10400C) was used as a nonspecific control. For each 1 mL of precleared lysate, ∼6 μg of antibody was added, and the sample was rotated at 4° overnight for ∼16 hr. Prewashed DynaBeads were added and the sample was rotated at 4° for 1 hr. Beads were collected by magnetic separation and washed with a series of buffers: low salt wash buffer [0.1% sodium dodecyl sulfate (SDS), 1% Triton X-100, 2mM EDTA, 20 mM Tris-HCl, 150 mM NaCl], high salt wash buffer (0.1% SDS, 1% Triton X-100, 2 mM EDTA, 20 mM Tris-HCl, 500 mM NaCl), and LiCl wash buffer (1% sodium deoxycholate, 1% NP-40, 1 mM EDTA, 10 mM Tris-HCl, 250 mM LiCl), 5 min each, and then washed in TE buffer once for 5 min. Antibody/protein was eluted in 200 μL of 50° elution buffer (1% SDS, 0.1 M NaHCO_3_); supernatant was removed, and a second 200-μL elution was performed. Cross-linking was reversed by addition of 20 μL of 5M NaCl to each 400-μL sample, and incubation at 65° for 4 hr. Then, 31 μL of PK solution (10 μL of 0.5M EDTA, 20 μL of 1 M Tris-HCl, pH 6.8, 1 μL of 20 mg/mL proteinase K) was added, and samples incubated at 45° for 1 hr. DNA was recovered by phenol/chloroform and isopropanol extraction. Before performing ChIP with adult chromatin extracts, we optimized conditions using chromatin prepared from synchronized L3 larvae and performed real-time PCR to assay genomic regions reported to be enriched/not enriched for H3K9me2 by modENCODE. We subsequently performed ChIP with synchronized adult hermaphrodites, as described.

DNA libraries were generated using the NEBNext ChIP-seq Library Prep Master Mix Set for Illumina (NEB #E6240) following the manufacturer’s protocol and using QIAGEN column purification. Library concentration and size were evaluated via Qubit fluorometric quantification (Invitrogen) and on a Bioanalyzer (Agilent Tech), respectively. DNA was sequenced at Cornell University on an Illuminar Highsequation 2500. In total, four ChIP DNA libraries were generated, with an average of ∼10.6 million unique mapped reads per library (see the section *Elevated H3K9me2 levels are observed in* him-8 *mutants*).

### mRNA-seq

Gonad dissection protocol was adapted from (Wang *et al.* 2009). Gonads were dissected from hermaphrodites at L4 stage + ∼18−24 hr. Animals were washed in “red PBS” (1× PBS + 1mM aurin tricarboxylic acid, an RNAse inhibitor) and dissected in red PBS with 0.2 mM Levamisole to immobilize worms. A total of 10−20 animals were dissected at a time; ∼15 gonad arms were collected (cutting at the spermatheca) per set of dissected animals. Dissected gonads were placed into Trizol (Invitrogen) on ice. Dissected material was stored for as many as several days at −80°. RNA was isolated following the standard Trizol protocol. Messenger RNA was isolated using NEBNext Poly(A) mRNA Magnetic Isolation Module (NEB#7490). Libraries for RNA-seq were constructed with the Illumina TruSeq RNA Sample Preparation Kit v2 (cat no.: RS-122-2001) following the protocol provided. Subsequent steps were the same as for ChIP-seq. In total, four mRNA libraries were generated; an average of 54 millions reads aligning perfectly to the *C. elegans* genome (see *Impact of H3K9me2 loss on the gonad transcriptome*) was obtained per library. The Pearson correlation coefficients for biological replicates of *met-2;him-8* and *him-8* are 0.87 and 0.97, respectively.

### Analysis of DNA sequence data

ChIP-seq data were mapped to the genome using Bowtie (v1.0.0) or BWA (v0.7.7) aligner (Langmead *et al.* 2009; Li and Durbin 2009); only those reads mapping uniquely to a single position in the genome were analyzed further. Peaks were called using MACS2 (v2.0.10.20130306-beta) (Zhang *et al.* 2008) with input as control (sample to input) or one of ChIP-seq datasets as control (sample to sample). BEADs analysis (v1.1) then was used to normalize the data as described (Cheung *et al.* 2011; Vielle *et al.* 2012). Two biological replicates were averaged after BEADs standardization, and H3K9me2 distribution relative to important genomic features was characterized with the use of CEAS (v1.0.2) (Shin *et al.* 2009). Bedtools (v2.17.0) (Quinlan and Hall 2010) was used to assist in finding differential peaks. R (v3.1.1) was used to plot the results. RNA-seq data were mapped to the genome using STAR (v2.3.0) aligner (Dobin *et al.* 2013). Cufflink (v2.2.1) and Cuffdiff (v2.2.1) were used to call RPKM values and determine which transcripts are present at significantly different levels in the various datasets (Trapnell *et al.* 2013). Bedtools (v2.17.0) was used to find genes located at H3K9me2-enriched sites. The IGV (v2.3.36) genome browser was used to visualize data (Robinson *et al.* 2011). All sequencing data have been submitted to the GEO database at NCBI as series GSE67030.

We took two approaches to identify repeat regions.

1. We screened the *C. elegans* genome (version WS 220) for annotated repeats listed in Repbase (version 20140131, www.girinst.org) using RepeatMasker (version open-4-0-5, repeatmasker.org, search engine RMBlast). This procedure identified seven types of repetitive sequence, classified by Repbase as Retro elements, DNA transposons, Helitrons, unclassified interspersed repeats (unassigned repeats), satellite sequences, simple repeats, and low complexity repeats. We then screened the H3K9me2-enriched sequences identified in *fer-1;him-8* and *fer-1* ChIP experiments using the same method.
2. We downloaded the Wormbase repetitive DNA data set, which includes additional repetitive sequences not annotated by Repbase (listed as “other” in Table S1), and compared these sequences to our ChIP-seq data sets using Bedtools.

### Quantitative real-time PCR

Real-time PCR was used to assay H3K9me2 ChIP results for select sites before deep sequencing. Real-time PCRs were performed with the Bio-Rad CFX Connect Real-Time PCR Detection System and RT-PCR reagent (Invitrogen). Primers used are listed in Table S3.

### Data availability

ChIP-seq and gene expression data are available at GEO with the accession number GSE67030. Table 1 lists the distribution of H3K9me2 enrichment on different classes of repetitive sequence. Table S2 summarizes the developmental phenotype associated with *wago-1;met-2* double mutants and controls. Figure S1 contains representative examples of H3K9me2 labeling, as detected by indirect immunofluorescence, of all relevant strains used in the ChIP-seq experiments. Figure S2 compares our adult ChIP-seq data with adult data from the modENCODE project for select sites. Figure S3 compares our adult data with germline-specific ChIP-seq data from the modENCODE project for select sites. Figure S4 contains data to indicate the low abundance of H3K9me2 in isolated gonads is not an artifact of the dissection process. Figure S5 demonstrates that H3K9me2 labeling, as detected by indirect immuofluorescence, is normal in MAGO12 males.

**Table 1:** Most sites of H3K9me2 enrichment are common to *fer-1;him-8* and *fer-1* adults

LG	H3K9me2 Elevated	H3K9me2 Reduced	H3K9me2 Absent*^a^*	*de novo* Sites*^b^*
I	867	320	0	73
II	649	255	1	57
III	752	341	0	60
IV	622	308	2	67
V	653	483	2	60
X	409	144	3	31
Total	3952	1851	8	348

>99.9% of the ∼8200 sites of H3K9me2 enrichment detected in *fer-1* adult hermaphrodites are also detected in *fer-1;him-8* adult hermaphrodites (see text). A large proportion of these sites are either elevated or reduced in *fer-1;him-8* compared with *fer-1* controls: ∼48% of sites are enriched at a significantly greater level in *fer-1;him-8*, and ∼23% of sites are enriched at a significantly reduced level in *fer-1;him-8*. Enrichment at most of the remaining ∼2400 sites (not listed here) is detected at an equivalent level in *fer-1* and *fer-1;him-8*. H3K9me2, histone H3 lysine 9 dimethylation

a Less than 0.1% of the sites enriched for H3K9me2 in *fer-1* lack that enrichment in *fer-1;him-8*.

b A number of *de novo* sites of H3K9me2 enrichment are detected in *fer-1;him-8*.

## Results

### Elevated H3K9me2 levels are observed in *him-8* mutants

To identify sites of elevated H3K9me2 on unsynapsed X chromosomes, we performed ChIP-seq analysis of control adult hermaphrodites (*fer-1*) and adult hermaphrodites with an X chromosome synapsis defect (*fer-1;him-8* mutants). The *him-8* mutation disrupts X chromosome pairing and synapsis (Hodgkin *et al.* 1979; Phillips *et al.* 2005) and the *fer-1(ts)* mutation impairs spermatogenesis, thereby preventing embryo production (L’Hernault *et al.* 1988). As shown in Figure S1, sites of high H3K9me2 enrichment are detected in *fer-1;him-8* meiotic nuclei via indirect immunofluorescence as expected given the unsynapsed X chromosomes. Meiotic germ cells comprise a substantial proportion (∼25%) of nuclei in the adult hermaphrodite (*e.g.*, see Qiao *et al.* 1995), and therefore we expected the H3K9me2 signal on meiotic X chromosomes to add substantially to the total X chromosome reads in our ChIP-seq dataset. We performed ChIP-seq on chromatin extracts prepared from synchronized *fer-1* and *fer-1;him-8* adult hermaphrodites raised at the *fer-1* restrictive temperature, 22° (see the section *Materials and Methods*). We performed two biological replicates and obtained an average of ∼10.6 million unique reads mapped to the *C. elegans* genome per ChIP library for the *fer-1* and *fer-1;him-8* libraries (see the section *Materials and Methods*).

**Figure 1:**
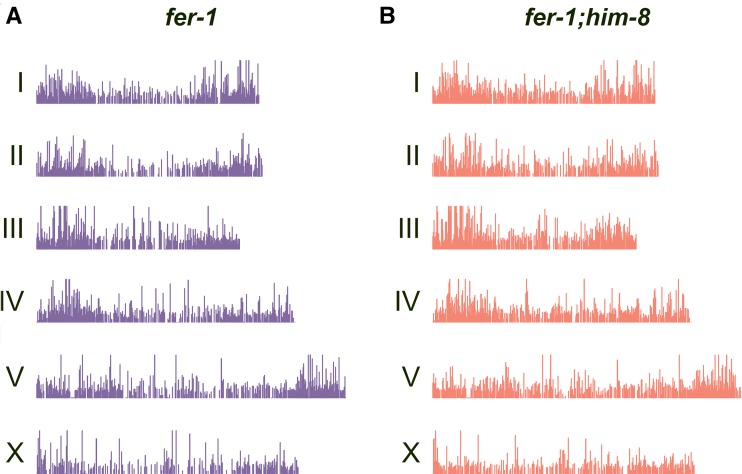
Histone H3 lysine 9 dimethylation (H3K9me2) enrichment across the genome of *fer-1* control and *fer-1;him-8* adult hermaphrodites. Bars represent regions that are enriched for H3K9me2 compared with input in staged (A) *fer-1* control and (B) *fer-1;him-8* adults. The *fer-1(ts)* allele impairs spermatogenesis and therefore prevents production of embryos; the *him-8* allele impairs X chromosome pairing and synapsis in XX animals. X-axis reflects chromosome length; Y-axis reflects the relative amount of H3K9me2 detected at each position.

We observed a low level of reads along the length of each chromosome and sequence enrichment (relative to input) at ∼8200 regions across the *fer-1* genome. Data are summarized in Figure 1. This H3K9me2 enrichment pattern is similar to those previously observed by the modENCODE consortium in embryos and L3 larvae (Gerstein *et al.* 2010; Liu *et al.* 2011) (Figure S2). The similarity of H3K9me2 distribution among developmental stages was striking, given that embryos and L3 larvae comprise mostly somatic cells whereas adults contain a large germ line. This finding suggested that either the H3K9me2 pattern is similar in germline and soma, or the level of H3K9me2 in the germ line is much lower than in the soma. H3K9me2-enriched regions tended to include repeated sequence (see Figure S2): 92% of the individual sequence reads at these enriched regions contained repetitive sequence, and 56.8% of the total sequence recovered is repetitive (Table S1). In contrast, repeated sequence comprises ∼19.6% of the total genome (www.wormbase.org annotation WS220). This 19.6% includes all repeated sequences, ranging from simple repeats (as small as 11 nucleotides) to large repeated regions of ∼45 kb. When we examined the distribution of H3K9me2 relative to different classes of annotated repeat sequence, we found that interspersed repeats, including DNA transposons, helitrons, and retroelements, are substantially enriched for H3K9me2 marks (Table S1).

**Figure 2:**
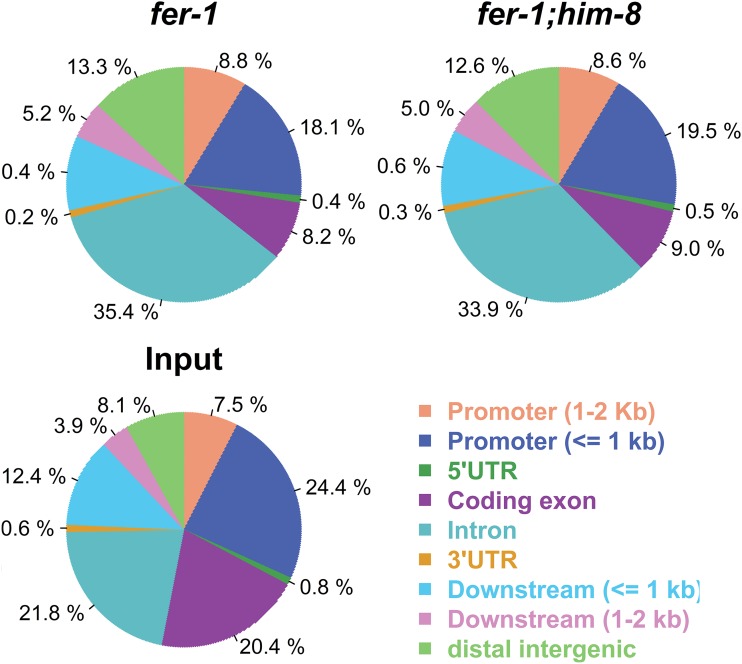
Distribution of histone H3 lysine 9 dimethylation (H3K9me2) chromatin immunoprecipitation sequencing reads relative to genome features. Pie charts indicate the percentages of H3K9me2-enriched regions that are located in the (predicted) promoter regions, 5′ untranslated regions (UTRs), coding exons, introns, 3′ UTRs, and intergenic regions. We note that promoter and 5′ UTR regions are as predicted in the current *C. elegans* genome database (WS 220) and in many cases are only approximate. Input is included for comparison. Overall, H3K9me2 is enriched in introns and depleted in exons with respect to input; other regions are similar to input. Each category is mutually exclusive.

**Figure 3:**
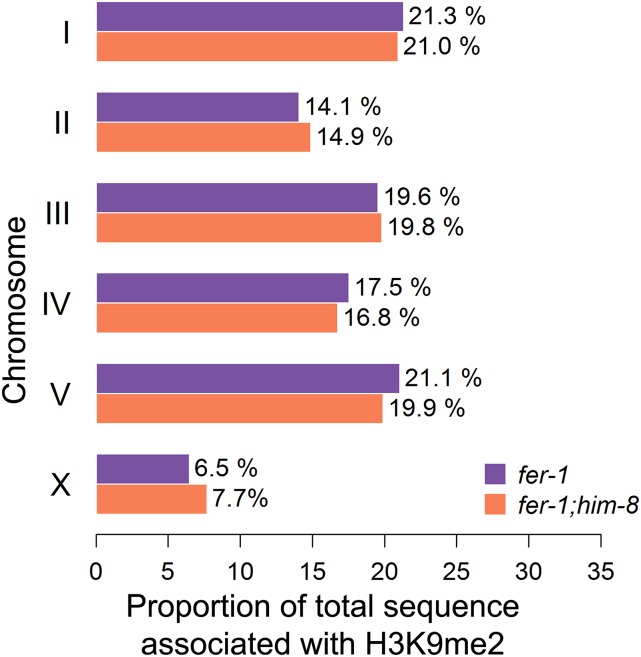
X-chromosomal distribution of histone H3 lysine 9 dimethylation (H3K9me2) enrichment is elevated in *fer-1;him-8*. The total length of H3K9me2-enriched DNA in the genome was calculated, and the proportion of this enriched sequence present on each chromosome was determined. In the *fer-1* control, 6.5% of the enriched sequence was on the X. In *fer-1;him-8*, 7.7% of total enriched sequence was on the X, an increase of 18% compared with control.

Analysis of H3K9me2 distribution relative to gene features indicates overall enrichment in introns and depletion in exons, reflecting the presence of interspersed repeats in many introns and their absence from exons (Figure 2 and Figure S2). H3K9me2-enriched regions are underrepresented on the X chromosome relative to autosomes, as illustrated in Figure 3 where the proportion of total H3K9me2-enriched sequence in the genome is represented on a per chromosome basis (see the section *Materials and Methods*). This enrichment pattern may reflect (at least in part) the relative paucity of repetitive sequences on the X: 12.9% of X chromosomal sequences are repetitive compared with 18.9–24% of autosomal sequences (data from wormbase.org).

**Figure 4:**
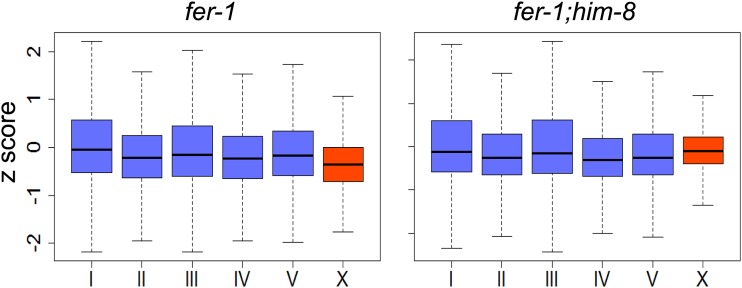
Total histone H3 lysine 9 dimethylation (H3K9me2)-associated X-chromosome sequence read count is elevated in *fer-1;him-8*. Plots represent the H3K9me2 signal on each individual chromosome relative to the average autosomal signal. Plotted data have been corrected for biased input using the BEADS method (Cheung *et al.* 2011). Each box represents z-scores ranging from the 25th to 75th percentile; line indicates the mean value; whiskers indicate the 2.5th and 97.5th percentiles. Average number of X chromosomal reads is greater and variation is reduced in *fer-1;him-8* relative to *fer-1* controls.

When we compared the H3K9me2 distribution in control and *him-8* mutants, we observed a similar general distribution of marks across the genome and correlation with repetitive sequences (Figure 1 and Figure S2). In the *him-8* dataset, 92% of enriched regions contained at least one repetitive sequence, and 53.3% of the enriched sequence was repetitive (Table S1). When we compared the *total* number of sequence reads on the X chromosome with the average number of total sequence reads on the autosomes (see the section *Materials and Methods*), we detected a 20% increase in total X chromosomal reads, relative to autosomal reads, in *him-8* adult hermaphrodites compared with controls (Figure 4). These reads are distributed broadly across the chromosome. When we specifically compared regions that are *enriched* for H3K9me2 on a per-chromosome basis, we see that the *proportion* of the total H3K9me2-enriched area on the X chromosome is 18% greater in *him-8* relative to controls, whereas the proportion of H3K9me2-enriched area on the autosomes decreases (Figure 3). Examining individual autosomes, the proportion of total H3K9me2-enriched area decreases or remains nearly unchanged on each autosome except for chromosome II; chromosome II behaved anomalously, with an ∼5% increase in the proportion of H3K9me2 coverage relative to controls (Figure 3). Overall, these data indicate an increase in X-linked H3K9me2 marks in *him-8* hermaphrodites.

**Figure 5:**
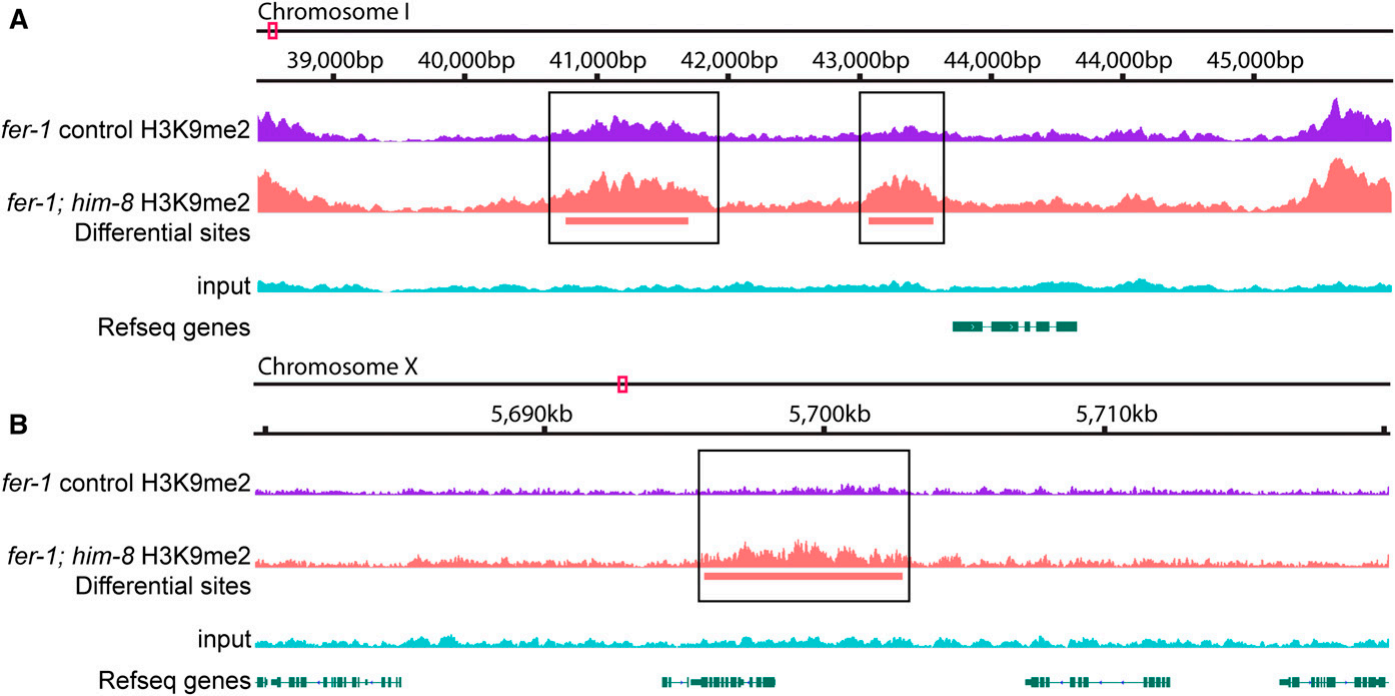
Examples of differential histone H3 lysine 9 dimethylation (H3K9me2) accumulation in the *fer-1* *vs.* *fer-1;him-8* genome. Tracks represent *fer-1* control H3K9me2, *fer-1;him-8* H3K9me2, differential sites, input reads, and annotated genes (Refseq). For H3K9me2 tracks, the average of two independent biological replicates is shown as normalized total reads. Representative genome browser tracts show differential sites that (A) have a greater signal in *fer-1;him-8* than in *fer-1* or (B) have a *de novo him-8−*specific site of enrichment. Differential regions are boxed. Y-axis scale reflects the number of reads, ranging from a minimum of 0 to a maximum of ≥ 120.

Although the *fer-1* and *fer-1;him-8* datasets are similar, we observed differential enrichment at many sites. Overall, ∼99.9% of the enriched sites present in *fer-1* were also enriched in *fer-1;him-8*. We were surprised to observe a statistically significant increase in enrichment at approximately 50% of these sites distributed across the genome (Table 1). A statistically significant reduction in enrichment was observed at approximately 25% of the sites, and enrichment at the remainder of the sites was not significantly different in the two data sets (Table 1). We also identified a small number of *de novo* sites that are present specifically in *him-8*. To do so, we first identified sites that might be unique to *fer-1* or *fer-1;him-8* using MACS2.1 (see the section *Materials and Methods*), and then visually inspected all sites that had been called as unique to one genotype to validate the peak calling. This approach identified a relatively small set of 348 *him-8*−specific sites distributed across the genome (Table 1 and Figure 5). These *de novo* sites tend to be associated with repetitive sequences, although to a slightly lower degree than the conserved sites: 85% of the 348 *de novo* regions include at least one repetitive sequence, and repetitive sequences comprise 41% of the *de novo* enrichment. The net effect of the observed differential H3K9me2 deposition is a general increase in H3K9me2 level in *him-8*. Taken together, our data are consistent with a global increase in H3K9me2 across the *him-8* hermaphrodite genome, and a further enhanced increase on the unsynapsed X chromosomes.

### Evaluation of H3K9me2 levels in gonads *vs.* whole animals

**Figure 6:**
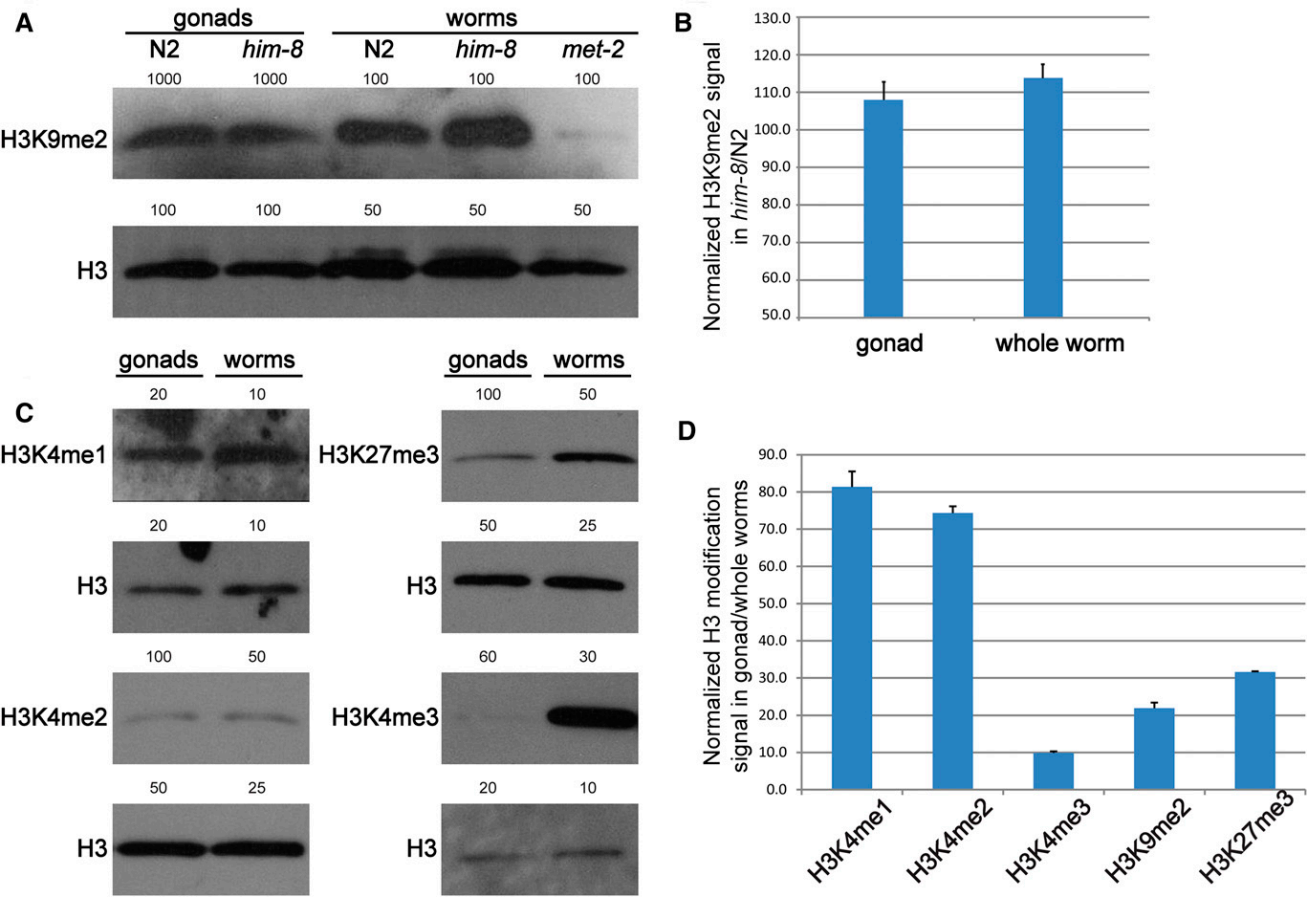
Histone H3 lysine 9 dimethylation (H3K9me2) abundance is elevated in *him-8* and low in the germline relative to the soma. (A, C) Representative protein blots probed with pan-H3 antibody and antibodies against several modified forms of histone H3. The number of whole adult hermaphrodites or dissected adult hermaphrodite gonad arms used to prepare extract for each lane is indicated. As the *C. elegans* gonad is primarily germ line, most of the histone H3 in gonad extracts is from germ cells (see *Impact of H3K9me2 loss on the gonad transcriptome*). Aliquots of a single extract were probed for total H3 and an individual H3 modification. Signal was quantified and the ratio of H3 modification to pan-H3 signal was calculated to determine the normalized value for each modification. (B) Histogram represents the ratio of normalized H3K9me2 signal in *him-8* relative to wild-type (N2) whole animals and isolated gonads. H3K9me2 signal is greater in *him-8* whole animals and gonads. (D) Histogram represents the ratio of normalized signal in isolated gonads relative to whole animals. Note that the Y-axis scale is different in B and D. Data represent three biological replicates for H3K4me1 and two biological replicates for each other mark (see the section *Materials and Methods*). Error bars, SEM.

To validate our ChIP-seq data analysis, we evaluated H3K9me2 levels in *fer-1* and *fer-1;him-8* adults and isolated adult gonads by performing protein blots. Using an antibody reported to detect H3K9me2 reliably on protein blots (Antibody Validation Database, http://compbio.med.harvard.edu/antibodies/), we detected a greater level of H3K9me2 relative to pan-H3 in *fer-1;him-8* whole worms and dissected gonads than in *fer-1* control whole worms and dissected gonads (∼114% and ∼107%, respectively; Figure 6). These results confirm the ChIP-seq finding that H3K9me2 levels are elevated in *him-8* mutants.

The H3K9me2 enrichment detected on *him-8* X chromosomes was less pronounced than we had expected *a priori* based on indirect immunofluorescence studies. We considered that the moderate X chromosomal H3K9me2 enrichment in *fer-1;him-8* might indicate that H3K9me2 marks are under-represented in germline chromatin. In this case, germ line contribution to the ChIP-seq signal would be lower than expected based on the proportion of meiotic nuclei present in adults. We analyzed the protein blot data to assess whether H3K9me2 marks were less prevalent in gonads than in whole animals. For both *fer-1* and *fer-1;him-8* animals, we detected H3K9me2 at a lower level relative to pan-H3 in isolated gonads than in whole animals (∼20%; Figure 6). Hence, this mark is underrepresented in the germline compared to the soma. Given this result, it appears that only ∼20% of the H3K9me2 signal detected by H3K9me2 ChIP-seq is germline. Therefore, if the 20% increase in X-linked reads we detected in *him-8* mutants results primarily from an increase in the germ line, then this would represent a substantial increase in germline X-linked H3K9me2. A low abundance of H3K9me2 in the germ line is consistent with recent modENCODE data examining H3K9me2 in germ cell nuclei isolated from young adult hermaphrodites (Ho *et al.* 2014); these data indicate a relatively even distribution of H3K9me2 marks across the genome compared with whole-animal data (Figure S3).

We included a *met-2(0)* (null) mutant as a negative control in our assays. Despite using a highly specific antibody, we detected a low H3K9me2 signal in protein extracts prepared from *met-2(0)* whole worms (Figure 6) and variably detected an extremely low signal in extracts prepared from dissected gonads (data not shown). As previously stated, H3K9me2 is not detected in gonads via indirect immunofluorescence (Bessler *et al.* 2010). Given these apparently conflicting results, we hypothesize that either *met-2(0)* mutants contain residual H3K9me2 not detected by IF or the anti-H3K9me2 antibody cross-reacts weakly on protein blots with other marks, *e.g.*, H3K9me3, that are unaffected in *met-2* mutants. We note that *met-2(0)* embryos have been reported to contain a residual level of H3K9me2 as detected by mass spectrometry (Towbin *et al.* 2012).

We evaluated several other histone H3 modifications and observed that each is underrepresented in the germline relative to whole animals, although to widely varying degrees (Figure 6). These findings reveal two aspects of chromatin regulation in the *C. elegans* germ line: the abundance of certain histone modifications is (much) lower than in the soma overall, and the ratio of different modifications to each other is very different than in the soma overall. Whole-animal data obviously represent an averaging of signals from numerous tissues, and our results emphasize this point for the germ line.

As a control, we considered that tissue dissection might trigger the removal of N-terminal histone tails, thus reducing our ability to detect certain marks. To investigate this idea, we performed a series of controls where we dissected adults, let the tissues sit in buffer 0, 1, 2, or 3 hr, and then analyzed the dissected tissue via protein blot. The histone signal did not decrease over time, and cleavage products did not appear, therefore we conclude that the low signal in dissected gonads is not an artifact of the dissection process (Figure S4).

### Impact of H3K9me2 loss on the gonad transcriptome

To evaluate the effect of H3K9me2 on the germline transcriptome, we isolated mRNA from *him-8* and *met-2*; *him-8* adult hermaphrodite gonads and performed deep sequencing (mRNA-seq; see the section *Materials and Methods*). We estimate that 90–95% of the nuclei in the dissected gonad arms are germline (∼325 germ cells per gonad arm *vs.* 10 somatic sheath cells and ≤24 somatic spermathecal cells; see www.wormatlas.org, Qiao *et al.* 1995). In addition, the hermaphrodite germ line generates high levels of maternal mRNA for inclusion in oocytes, which will increase the germline contribution to the steady state transcript level in the gonad. Therefore, gonad mRNA-seq data primarily reflect the germline transcriptome. We note that *met-2(0)* mutants have a mortal germline (Mrt) phenotype: when maintained as homozygotes for many generations, they progressively lose their germline and eventually become sterile (Andersen and Horvitz 2007; Bessler *et al.* 2010). In our experiments, we were interested in examining the effect of H3K9me2 loss over the short term; therefore, we used *met-2(0)* animals recently derived from a balanced strain, which were fertile and did not exhibit a severely reduced germ line. Two biological replicates were performed wherein mRNA was isolated from separate sets of dissected gonads and used to generate libraries for sequencing (see the section *Materials and Methods*).

**Figure 7:**
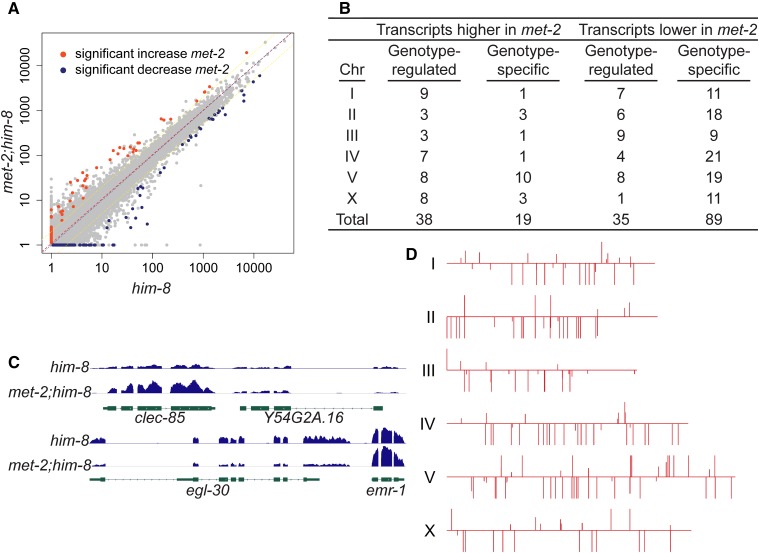
The effect of histone H3 lysine 9 dimethylation (H3K9me2) loss on the adult hermaphrodite gonad transcriptome. (A) Scatter plot represents the transcriptome data from *him-8* gonads *vs.* *met-2;him-8* gonads. Transcripts present at a statistically different level are highlighted in red if more highly expressed in *met-2;him-8* and blue if more highly expressed in *him-8*. X- and Y-axis scale is expressed in RPKM (see text). (B) The table lists the number of genes on each chromosome with transcripts that are differentially abundant in *met-2*; *him-8* *vs.* *him-8* gonads. These represent the highlighted transcripts in (A). (C) Screen shots of representative differential transcripts: *clec-85* transcripts are elevated in *met-2;him-8* and *egl-30* transcripts are reduced in *met-2;him-8*. Transcripts from adjacent genes, Y54G2A.16 and *emr-1*, are statistically unchanged. (D) Chromosomal distribution of genes whose transcripts are differentially abundant in *met-2;him-8* *vs.* *him-8*. Lines extending upward indicate transcripts that are more abundant in *met-2*; *him-8*, and lines extending downward indicate transcripts that are less abundant in *met-2;him-8*. Longer lines represent “genotype-specific” genes whose transcripts are detected in one genotype and not the other. Shorter lines represent genotype-regulated genes whose transcripts are more abundant in one genotype than the other; their length is proportional to the log_2_ value of the fold-change.

We compared the *him-8* and *met-2;him-8* gonad transcriptomes to each other. We calculated the RPKM (Reads Per Kilobase of transcript per Million mapped reads) for each transcript in our mRNA-seq datasets. For purposes of comparing the *him-8* and *met-2;him-8* transcriptomes, we considered transcripts present at a relatively permissive cut-off value of >0.5 RPKM. A transcript was defined as present at a significantly different level in *him-8* *vs.* *met-2;him-8* if the Cuffdiff q-value was <0.05 (see the section *Materials and Methods*). The vast majority of transcripts were present at statistically similar levels in both datasets; however we did identify a set of 181 differentially expressed transcripts (Figure 7) that fall into two classes. Transcripts from 108 genes were detected in only one genotype (RPKM = 0 in the other genotype); we refer to these as genotype-specific transcripts. Transcripts from 73 genes were detected in both genotypes, but at significantly different levels; we refer to these as genotype-regulated transcripts. The log_2_ fold-change of genotype-regulated transcripts ranged from 5.3 (40-fold) to 0.96 (1.9-fold). These 181 differentially regulated genes are distributed across the genome (Figure 7D).

We compared our gonad transcriptome data with the results of a previously published serial analysis of gene expression (SAGE) study of gonad-expressed mRNA (Wang *et al.* 2009). In this study, soma-expressed transcripts were identified by comparison with *glp-4* germ cell-less mutants; transcripts absent from the *glp-4* mutants were deemed germline-specific. This approach identified transcripts from 4699 genes as present in at least one copy in 100,000 reads; using an average transcript size of 1.3 kb, this means the low detection cut-off was approximately equivalent to an average RPKM of 7.7. Our *fer-1* dataset contains 6869 genes with an RPKM of >7.7. As expected, these 6869 genes include >91% of the total and 99.9% of the germline-specific genes identified in the SAGE analysis. [Transcripts from the remaining germline-expressed genes identified in Wang *et al.* (2009) were also expressed in our dataset, but at an RPKM of <7.7.] Among the 181 transcripts we identified as differentially regulated in *met-2* *vs.* *met-2;him*-8, 40 were also identified in the SAGE dataset, and 17 of these were identified as germline-specific. The other 141 genes in are expressed at a low level (RPKM <5) in our data, which is presumably why they are absent from the SAGE dataset.

We also examined the identity of differentially expressed genes with respect to known and hypothesized gene function by performing Gene Ontology (GO) analysis. Using the DAVID algorithm (Huang *et al.* 2009), we found that a large proportion of the differentially expressed genes (123/181) are not associated with a GO molecular function term. The remaining 58 genes are associated with a variety of GO molecular function terms and are not enriched for any particular term.

### Distribution of H3K9me2 enrichment relative to germline-expressed genes

**Figure 8:**
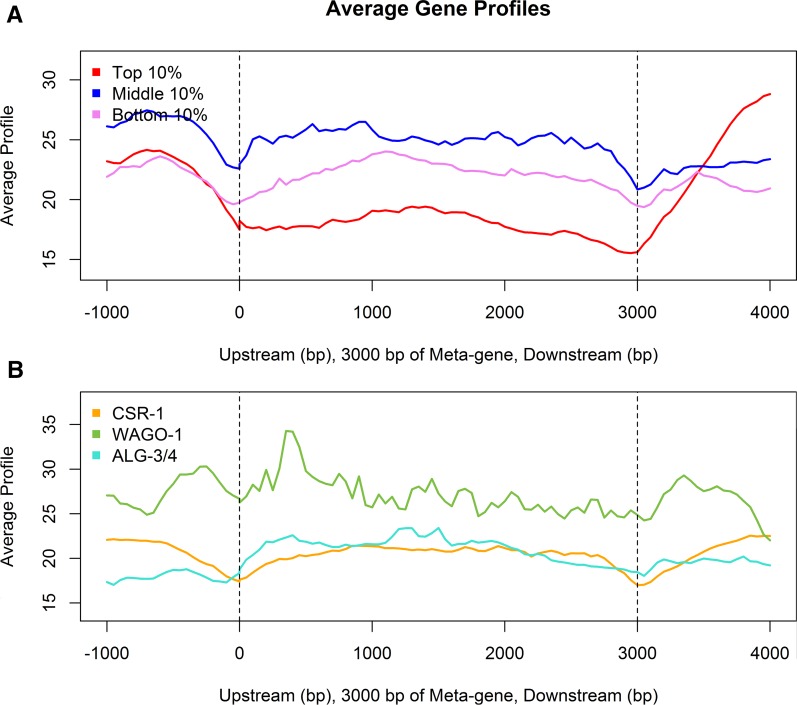
Meta-gene analysis of the adult hermaphrodite histone H3 lysine 9 dimethylation (H3K9me2) profile across gonad-expressed genes and predicted Argonaute targets. Gene expression data are from our RNA-seq analysis of *him-8* gonads. The meta-gene includes 1 kb of sequence upstream of the transcription start site (0), a 3 kb averaged coding region, and 1 kb downstream of the transcription termination site (3000). (A) The average H3K9me2 enrichment signals of the top 10%, middle 10%, and bottom 10% of expressed genes in the *him-8* adult hermaphrodite gonad. (B) The average H3K9me2 enrichment of predicted CSR-1, WAGO-1, and ALG-3/4 target genes (Claycomb *et al.* 2009; Conine *et al.* 2010; Gu *et al.* 2009). The 96 most highly represented WAGO-1 targets, used in this analysis, are substantially enriched for H3K9me2 across the entire meta-gene.

We performed meta-gene analysis (Shin *et al.* 2009) to evaluate the distribution of H3K9me2 marks with respect to transcript level in *him-8* gonads. H3K9me2 levels were relatively low within a 2-kb window flanking transcription start and stop sites and higher in introns (Figure 8A) as observed previously for whole embryos and L3 larvae (Figure 8A) (Gerstein *et al.* 2010; Liu *et al.* 2011). In addition, H3K9me2 levels were lowest at genes with a relatively high transcript level (Figure 8A) and were present at a similar level on genes expressed at a moderate or low level. This pattern is consistent with other studies reporting that H3K9me2 marks do not strictly correlate with transcriptional inactivity and are often present within the body of genes located within heterochromatic regions (Yasuhara and Wakimoto 2008; Ho *et al.* 2014; West *et al.* 2014).

We hypothesized the loss of MET-2 activity in the *him-8* background might disproportionally impact the level of X-linked transcripts. If H3K9me2 marks correlate with reduced transcription of nearby/adjacent genes, then we might expect to see a significant increase in transcript levels in the *met-2(0)* mutant where those marks are absent, and this pattern might be especially obvious in the *him-8* background where H3K9me2 marks are elevated. Instead, only a minority (57 of 181, 31%) of the differentially expressed transcripts were elevated in *met-2* mutants (Figure 7A). This pattern is particularly striking for the genotype-specific genes: 89 of 108 (82%) of them are off in *met-2* mutants (lacking H3K9me2) and only 19 of 108 (18%) of them are off in the control (containing H3K9me2). A total of 23 of the 181 differentially regulated transcripts are X-linked. Of these, 11 are elevated in the *met-2* background and 12 are reduced. Hence, there is no clear pattern with respect to H3K9me2 marks and germline transcript level.

We also evaluated H3K9me2 enrichment, as detected by ChIP-seq, relative to germline-expressed genes. We first compared the H3K9me2 enrichment we detected in *fer-1* adults with the H3K9me2 enrichment in adult germ lines reported by Ho *et al.* (2014) to see whether the latter data set contained germline-specific H3K9me2 sites that had not been detected in our whole animal data set. To make this comparison, we analyzed the germ line data of Ho *et al.* (2014) in the same way we had analyzed our whole animal data. This analysis revealed ∼400 sites of H3K9me2 enrichment (as opposed to ∼8200 sites in whole animals), each of which we had detected as enriched in our *fer-1* whole animal dataset, and 98% of which contained repetitive sequence. These results are consistent with our finding that H3K9me2 abundance is low in the germ line. Because these data did not reveal germline-specific sites of H3K9me2 accumulation, we then used our whole genome dataset to ask whether the few *de novo* sites of H3K9me2 accumulation in *fer-1;him-8* hermaphrodites correlated with genes whose transcript levels were altered in *met-2* mutant germ cells. To do so, we compared the differential H3K9me2 peaks detected in our ChIP-seq experiments with differentially expressed genes detected in our mRNA-seq experiments by calculating the correlation coefficients between data sets (see the section *Materials and Methods*). We identified H3K9me2-enriched regions located within genes or adjacent to genes within 500 bp of the predicted transcription start site. This analysis revealed 40 H3K9me2-associated genes among the 181 genes differentially expressed genes in *met-2(0)* and control animals. We did not observe a correlation between MET-2 activity (*i.e.*, H3K9me2) and transcript level among these genes: 16 were expressed more highly in *met-2(0)* animals and 24 were expressed more highly in *met-2(+)* animals.

### H3K9me2 distribution with respect to Argonaute targets

As described previously, H3K9me2 distribution in the germ line, as detected by IF, depends on activity of CSR-1 Argonaute and proteins required for production of CSR-1−associated 22G RNAs, EGO-1, DRH-3, and EKL-1 (She *et al.* 2009). We were interested in evaluating the extent to which H3K9me2 marks might correlate with putative targets of Argonaute pathway activity. Consequently, we evaluated the H3K9me2 profile at putative CSR-1, WAGO-1, and ALG-3/4 targets as reported in the literature (Gu *et al.* 2009; Claycomb *et al.* 2009; Conine *et al.* 2010) (Figure 8B). The H3K9me2 profile is relatively low for CSR-1 and ALG-3/4 targets, similar to levels observed for germline-expressed genes. In contrast, the H3K9me2 profile for WAGO-1 targets is notably greater overall and particularly elevated in sequences flanking the transcription start site and immediately downstream of the transcription termination site. WAGO-1 targets are reported to include repetitive elements, pseudogenes, intergenic regions, and some protein-coding genes (Gu *et al.* 2009). In our meta-analysis, we used the top 96 reported WAGO-1 targets, 95 of which are protein-coding genes (Gu *et al.* 2009); 67% (66/96) of these WAGO-1 target genes contain at least one repetitive region, although these regions are highly variable in size. We also analyzed supplemental data (Gu *et al.* 2009) to compare transposon and other repeat sequence targets of WAGO-1 to repeat sequence targets of H3K9me2. SiRNAs corresponding to many retrotransposons and DNA transposons, but not helitrons, were identified in association with WAGO-1. Consequently, a subset of repeat sequences appears to be commonly targeted by WAGO-1 and H3K9me2.

Previously, we surveyed the H3K9me2 distribution via immunolabeling of Argonaute single mutant males and observed a defect only in *csr-1* mutants (She *et al.* 2009). H3K9me2 distribution appeared normal in males carrying a mutation in *wago-1* or any of the other individual WAGO clade genes. Interestingly, the *ego-1(0)* and *csr-1(0)* phenotypes are not identical: in *ego-1(0)* mutants, H3K9me2 foci are absent from unsynapsed chromosomes; in *csr-1(0)* mutants, weak foci are visible on unsynapsed chromosomes, and ectopic foci are visible on synapsed chromosomes (Maine *et al.* 2005; She *et al.* 2009). EGO-1 is required for biogenesis of siRNAs that associate with either CSR-1 or the WAGOs (Gu *et al.* 2009), and we wondered whether redundant WAGO activity might impact H3K9me2 distribution. We decided to evaluate meiotic H3K9me2 in males that carry MAGO12 (Gu *et al.* 2009) to address this question. As shown in Figure S5, H3K9me2 distribution in first meiotic prophase nuclei appears normal in MAGO12 males.

To begin to investigate the relationship between H3K9me2 marks and WAGO-1 activity, we generated a *wago-1(0)*; *met-2(0)* double mutant and evaluated the developmental phenotype. *met-2* single mutants are reported to develop an Mrt germline defect over 18−24 successive generations (Andersen and Horvitz 2007; Bessler *et al.* 2010). In our hands, the brood size of *wago-1;met-2* double mutants is substantially reduced at 20° within ∼12 generations; however, the strain does not become sterile sooner than the 18−24 generations described for *met-2* (Andersen and Horvitz 2007; Bessler *et al.* 2010), hence the *met-2* Mrt phenotype does not appear to be enhanced.

To evaluate developmental defects, we grew the strains at 20°, picked mid-L4 stage hermaphrodites to fresh plates, and DAPI-stained them 48 hr later. As previously reported, *met-2* mutants contain a variety of germline defects at a very low level (Bessler *et al.* 2010). The most common germline defects we observed in our *met-2* strain were (1) presence of endomitotic oocytes (an Emo phenotype), (2) the presence of clumped meiotic nuclei, and (3) the presence of some morphologically abnormal (typically small) nuclei in the meiotic region. We observed these defects at a very low level in our *wago-1* strain (Table S2). The frequency of the Emo phenotype was reduced in *wago-1;met-2* compared with *met-2*, whereas the frequencies of nuclear clumping and morphologically abnormal nuclei were increased additively in the *wago-1;met-2* double mutant compared with each single mutant. These results suggest MET-2 and WAGO-1 may act in parallel to impact some aspects of germ line development.

The most prominent somatic phenotypic we observed is an egg-laying defect (animals filled with developing embryos) detected at a low level in all three strains (Table S2). This defect was observed at the L4 + 48-hr time point, but not at the earlier L4 + 24-hr time point. Animals were visually examined on the culture plate and after DAPI staining and judged to have an Egl defect if at least a double row of embryos was observed in the uterus. The phenotype is more penetrant in *wago-1;met-2* double mutants than in *met-2* and *wago-1* single mutants (Table S2); the effects appear to be additive, suggesting that MET-2 and WAGO-1 activity act in parallel to impact egg laying.

## Discussion

Our studies describe and compare the genome-wide distribution of H3K9me2 in adult *C. elegans* hermaphrodites with synapsed (*fer-1*) *vs.* unsynapsed (*fer-1;him-8*) X chromosomes. Immunolabeling assays consistently detect elevated H3K9me2 on unsynapsed chromosomes in *him-8* hermaphrodites, and we performed ChIP-seq to distinguish whether the H3K9me2 enrichment accumulates at *de novo* sites on the unsynapsed meiotic X or to an elevated degree at sites normally present on synapsed chromosomes. Our data reveal that H3K9me2 is enriched primarily at existing sites, and at very few *de novo* sites, in *him-8* mutants. This X-enriched signal is relatively modest, most likely because H3K9me2 is underrepresented in germ cells compared with whole animals as detected in our protein blot experiments. Nonetheless, we interpret the increased X chromosome H3K9me2 signal as an elevated abundance of H3K9me2 on meiotic X chromosomes and a redistribution of MET-2 activity toward the unsynapsed Xs. Unexpectedly, in addition to detecting an increase in the proportion of H3K9me2 associated with the X chromosome in *him-8* mutants, we also observed a net increase in H3K9me2 abundance at sites distributed across the genome. Moreover, this increase is not specific to the germ line, but instead present in (at least some) somatic tissues, as well.

To compare H3K9me2 levels on a set of chromosomes when synapsed *vs.* non-synapsed, we necessarily evaluated an example of MSUC. We chose to use *him-8* and evaluate the X chromosome because this is the best-studied situation in the literature. However, these choices raise two points. First, the effect of MSCI might be stronger than MSUC, both in terms of H3K9me2 abundance and effects on the transcriptome. It is possible that MSCI, as a naturally occurring process, causes a more robust increase in H3K9me2 level and has a more profound impact on the transcriptome than does MSUC, which arises in due to mutation or chromosomal duplication. Second, X-linked gene expression is limited in the *C. elegans* germ line relative to autosomal gene expression (Kelly *et al.* 2002; Reinke *et al.* 2000). It is possible that MSUC triggered by unsynapsed autosomes, such as chromosome V in *zim-2* mutants, would have a greater impact on the germline transcriptome. However, *zim* mutants are fertile and have essentially normal germline development (Phillips and Dernburg 2006), therefore it seems unlikely that transcript levels are radically altered in those mutant backgrounds.

Another consideration is the residual H3K9me2 present in *met-2(0)* mutants. Analysis of H3K9me2 in embryos via mass spectrometry detected a low abundance of H3K9me2 in *met-2(0)* embryo extracts, a result is consistent with our protein blot data reported here (Towbin *et al.* 2012). Therefore, our analysis of *met-2(0)* provides information about the transcriptome in germ lines with a very low abundance of H3K9me2 marks. If randomly distributed, these residual marks might be expected to have minimal impact. However, their distribution is not known. These other marks appear to depend on SET-25 activity, as H3K9me2 was not detected in *set-25*; *met-2* double mutants (Towbin *et al.* 2012). SET-25 also is responsible for H3K9me3, as these marks are eliminated in *set-25* mutants. One consequence of this dual activity is that we do not have a means to eliminate H3K9me2 marks entirely without also eliminating H3K9me3, and therefore we cannot easily test the impact of the SET-25-dependent H3K9me2 on the germline transcriptome.

Although primarily studied for its role in meiosis, *him-8* is known to interact genetically with a number of somatically expressed transcriptional regulators (Nelms and Hanna-Rose 2006; Sun *et al.* 2007). It is possible that HIM-8 impacts H3K9me2 level by modulating activity of one or more transcriptional regulators that, in turn, modulate expression of histone-modifying enzymes. As *him-8* mutants develop normally for the most part, we speculate that the elevated H3K9me2 level may be balanced out by elevated levels of other histone modifications, as well.

We find that germline abundance of H3K9me2 and several other histone marks is low relative to abundance in the soma overall. We also observed that the relative level of histone H3 modifications in germline *vs.* soma varies widely, although each of the marks we assayed is underrepresented in adult germ lines relative to adult soma. Although it is to be expected that histone marks might vary among tissues and cells, our observations are important for interpreting ChIP data in that they indicate the germline signal varies widely depending on the mark being assayed. The low H3K9me2 abundance in *C. elegans* is reminiscent of the situation in mouse, where primordial germ cells lose H3K9me2 (and other) marks as they undergo epigenetic reprogramming (Seki *et al.* 2005). The abundance of H3K9me2 marks remains low in adult testes compared with somatic gonadal cells (Deguchi *et al.* 2013). Similarly sweeping changes in histone modification are observed in the *C. elegans* germ lineage (P4 and its daughters, Z2 and Z3) as it forms in the embryo (Wang and Seydoux 2013; Kelly 2014). The abundance of many marks, including H3K9me2 and others tested here, is low in Z2 and Z3 compared with somatic embryonic nuclei (Schaner and Kelly 2006). Subsequent changes in histone modification pattern are observed as the germline develops and produces gametes, with dramatic increases in many histone activation marks (reviewed by Van Wynsberghe and Maine 2013). The general paucity of histone H3 modifications that we detect in the adult germline may reflect on-going mitosis and differentiation in the adult germline, which is absent in the adult soma, and gamete-specific patterns of chromatin regulation important for meiotic events and/or early embryogenesis.

The pattern of H3K9me2 accumulation across the adult XX genome correlates positively with reported WAGO-1 Argonaute targets and negatively with reported CSR-1 Argonaute targets (Gu *et al.* 2009; Claycomb *et al.* 2009). A negative correlation with CSR-1 targets is consistent with our earlier observation of ectopic H3K9me2 accumulation in *csr-1*−null mutants (She *et al.* 2009). In other words, CSR-1 activity may *exclude* deposition of H3K9me2 marks at actively expressed chromatin regions. Moreover, current thinking is that CSR-1 functions in a mechanism to distinguish appropriate from inappropriate gene expression in the germ line (Seth *et al.* 2013; Wedeles *et al.* 2013). In this sense, CSR-1 is thought to be a positive regulator of germline gene expression. Positive correlation of H3K9me2 with WAGO-1 targets suggests H3K9me2 and WAGO-1 may act together or in parallel to regulate a common set of targets. In our analysis, we used the top 96 WAGO-1 targets identified based on recovery of 22G siRNAs by WAGO-1 co-IP; 95 of these targets are protein-coding genes (Gu *et al.* 2009). Therefore, WAGO-1 and H3K9me2 appear to have some common protein-coding gene targets. We also found that H3K9me2, like WAGO-1, preferentially modified retrotransposons and DNA transposons, although H3K9me2 marks also accumulate at helitrons and some other interspersed repeat sequences not targeted by WAGO-1.

Our genetic and immunolabeling data suggest that the two mechanisms act in parallel: *wago-1* and *met-2* developmental defects are additive, and H3K9me2 deposition appears normally enriched on the X chromosome in *wago-1* (She *et al.* 2009) and MAGO12 meiotic male germ cells (this study). Studies in mouse suggest H3K9 methylation acts in parallel with other mechanisms, piRNA and DNA methylation, to silence certain transposons (Di Giacomo *et al.* 2013; Di Giacomo *et al.* 2014). In *C. elegans*, transposons are known to be variably targeted for silencing by the 21U (pi) RNA/PRG and/or 22G RNA/WAGO pathways and for H3K9 methylation (reviewed by Van Wynsberghe and Maine 2013; Ni *et al.* 2014). There is overlap among these mechanisms, for example the nuclear RNAi (NRDE) pathway targets H3K9me3 marks to retrotransposons (Ni *et al.* 2014). Our data provide further evidence of parallel regulatory pathways targeting a common set of transposons.

Although H3K9me2 was originally described as a “silencing” mark due to its association with heterochromatin (Bannister *et al.* 2001), substantial data have accumulated to indicate that H3K9me2 can be present on expressed genes. In *C. elegans* embryos, H3K9me2 is associated with both active and inactive genes in nuclear envelope-associated chromatin (Ikegami *et al.* 2010). In *Drosophila*, distinct patterns of H3K9me2 accumulation are observed across active genes located in heterochromatin domains (H3K9me2 was present) *vs.* active genes located in euchromatin domains (H3K9me2 was absent) (Yasuhara and Wakimoto 2008). This flexible H3K9me2 accumulation pattern is in contrast to many other marks whose accumulation correlates strongly with either an active (*e.g.*, H3K36me1) or repressed (*e.g.*, H3K27me3) state (Ho *et al.* 2014). Consistent with published data from *C. elegans* whole-animal RNA-seq analysis (Ho *et al.* 2014), our RNA-seq results do not indicate a strong correlation between H3K9me2 and transcript level. Although the most highly expressed genes tend to have a lower H3K9me2 level than other genes, this trend does not extend to genes expressed at a low-moderate level. The presence of H3K9me2 on active genes in *C. elegans* heterochromatic regions is consistent with the observations in *Drosophila*. In a recent study, Garrigues *et al.* showed that H3K9me2 is a mark of heterochromatin in *C. elegans* as in other organisms (Garrigues *et al.* 2014). Their results define heterochromatin as regions containing H3K9me1, H3K9me2, HPL-2 (Heterochromatin Protein 1 ortholog), and a relatively high density of repetitive sequences. Most of the H3K9me2 enrichment we detect appears to be heterochromatic as it is associated with repeat sequences.

Consistent with the idea that H3K9me2 serves another function in addition to or instead of transcriptional control, our data reveal only modest differences in the gonad transcriptome in *met-2(0)* mutants *vs.* controls. This finding is consistent with immunolabeling results obtained by Checchi and Engebrecht in their analysis of checkpoint surveillance (Checchi and Engebrecht 2011). When they evaluated RNAPII association with unsynapsed chromosomes via immunolabeling, they observed variable association of active RNAPII with the X chromosome in meiotic nuclei in both *him-8* and *met-2;him-8* hermaphrodites. The authors interpreted these results to mean that H3K9me2 was not responsible for limiting transcription in the unsynapsed X homologs. We do note that our data do not provide information about the *met-2* germline transcriptome after successive homozygous generations, and we hypothesize that the transcriptome shifts as fertility decreases.

Numerous alternative functions for H3K9 methylation have been proposed based on a variety of studies. For example, H3K9me2 at repetitive regions may promote a chromatin configuration to reduce unequal crossing-over between repetitive sequences located at different positions on the chromosome. Such a scenario is hypothesized for *Drosophila*, where double-strand breaks (DSBs) arising in heterochromatin are shifted outside of the heterochromatin domain before RAD51 can be recruited for homologous recombination repair (Chiolo *et al.* 2011). On the basis of colocalization studies, DSBs that occur in heterochromatin domains attract early recombination markers, and then are relocated outside of the heterochromatin domain prior to RAD51 recruitment and strand repair (Chiolo *et al.* 2011). Moreover, heterochromatin proteins themselves appear to prevent RAD51 association. A recent fine-structure analysis of recombination on *C. elegans* chromosome II did not detect a correlation between H3K9me2 density and recombination: an abrupt increase in recombination frequency on the chromosome arm did not correlate with a similar increase in H3K9me2 density (Kaur and Rockman 2014). However, *C. elegans* recombination is well known to occur more frequently on autosomal arms than in the chromosome centers, and this pattern correlates with the relatively high repetitive sequence density on arms compared with centers (Gerstein *et al.* 2010; Liu *et al.* 2011).

In mouse, H3K9me2 accumulation at pericentric heterochromatin appears to contribute to meiotic pairing and synapsis through its association with heterochromatin protein 1 (Takada *et al.* 2011). In *C. elegans*, where chromosomes are holocentric, H3K9me2 does not accumulate at centromeric heterochromatin (Gassmann *et al.* 2012). However, chromosome pairing is mediated by a single pairing center at the end of one arm of each chromosome, and each pairing center contains a number of short sequence motifs responsible for recruiting pairing proteins (*e.g.*, HIM-8) (Phillips *et al.* 2009). Although chromatin in the vicinity of the pairing center contains substantial H3K9me2, these marks do not accumulate at the recruitment motifs themselves (Liu *et al.* 2011). Consistent with nuclear envelope association of heterochromatic regions, pairing centers are brought together via interaction with HIM-8 and other pairing proteins at the nuclear envelope (Phillips and Dernburg 2006). While there is no direct evidence that H3K9me2 modulates pairing in *C. elegans*, there may be a relationship between recombination and H3K9me2 distribution because the chromatin factor, HIM-17, is required both for DSB formation and for robust H3K9me2 accumulation at certain sites (Reddy and Villeneuve 2004).

Another postulated role for H3K9 methylation is in positioning heterochromatin at the nuclear periphery in association with the nuclear envelope, an association thought to limit gene expression. Heterochromatin in the *C. elegans* genome is concentrated on the autosomal arms and left end of X—coincident with H3K9me2 and repetitive sequences—and these regions associate with the nuclear envelope via LEM-2 in a manner that depends on H3K9 methylation (Garrigues *et al.* 2014; Ikegami *et al.* 2010; Towbin *et al.* 2012). We note that LEM-2 binds preferentially to helitron sequences, a class of transposon also enriched for H3K9me2.

In summary, the accumulation of H3K9me2 on unsynapsed X chromosomes may function in multiple ways to promote germline processes. The paucity of *de novo* H3K9me2 sites on unsynapsed chromosomes suggests that the increased level of H3K9me2 does not play a unique role on those chromosomes. Instead, assembly of heterochromatin on unsynapsed chromosomes may require an elevated level of H3K9me2 to confer the same control over chromosomal processes as obtained on synapsed chromosomes. Future studies may reveal the basis for this difference, and how H3K9me2 marks regulate chromosomal processes during meiosis.

## Supplementary Material

Supporting Information
