# Supplementary material for: Enrichment of H3K9me2 on Unsynapsed Chromatin in Caenorhabditis elegans Does Not Target de Novo Sites
Source: G3 (Bethesda). 2015 Jul 8;5(9):1865–78. doi: 10.1534/g3.115.019828 (PMC4555223; doi:10.1534/g3.115.019828)
Supplement: Supporting Information [file supp_5_9_1865__index.html]

Enrichment of H3K9me2 on Unsynapsed Chromatin in Caenorhabditis elegans Does Not Target de Novo Sites — Supporting Information 

# Enrichment of H3K9me2 on Unsynapsed Chromatin in *Caenorhabditis elegans* Does Not Target *de Novo* Sites

## Supporting Information for Guo *et al.*, 2015

**Files in this Data Supplement:**

- Supporting Information - Figures S1-S5 and Tables S1-S3 (PDF, 633 KB)
- Figure S1 - H3K9me2 is enriched on the unsynapsed X chromosomes in *fer-1;him-8* mutants. (PDF, 241 KB)
- Figure S2 - Comparison of H3K9me2 distribution in our adult datasets with L3 larval and early embryo datasets from modENCODE. (PDF, 256 KB)
- Figure S3 - Comparison of H3K9me2 distribution in our adult whole animal datasets with isolated adult germ cell dataset from modENCODE. (PDF, 236 KB)
- Figure S4 - Histone signal does not degrade in dissected tissue. (PDF, 153 KB)
- Figure S5 - H3K9me2 is enriched on male the X chromosome in MAGO12 mutants during first meiotic prophase. (PDF, 168 KB)
- Table S1 - H3K9me2 tends to be enriched at repetitive DNA sequences. (PDF, 159 KB)
- Table S2 - Developmental defects observed in *wago-1, met-2,* and *wago-1;met-2* XX adults. (PDF, 167 KB)
- Table S3 - Oligonucleotide primers used for real-time PCR reactions. (PDF, 130 KB)
