# Supplementary material for: Enrichment of H3K9me2 on Unsynapsed Chromatin in Caenorhabditis elegans Does Not Target de Novo Sites
Source: G3 (Bethesda). 2015 Jul 8;5(9):1865–78. doi: 10.1534/g3.115.019828 (PMC4555223; doi:10.1534/g3.115.019828)
Supplement: Supporting Information [file supp_g3.115.019828_019828SI.pdf]

G3 · Genes | Genomes | Genetics

Enrichment of H3K9me2 on unsynapsed chromatin in *Caenorhabditis elegans* does not target *de novo* sites

Yiqing Guo*, Bing Yang*, Yini Li*, Xia Xu*§, and Eleanor M. Maine*

*Department of Biology, Syracuse University, Syracuse, New York, 13244

§ Current address: Xia Xu, Department of Biochemistry and Molecular Biology, SUNY Upstate Medical University, Syracuse, New York, USA

Gene Expression Omnibus (GEO) accession link: GSE67030

Corresponding author:

Eleanor M. Maine

Department of Biology / Life Science Complex

107 College Place

Syracuse, New York 13244

315-443-9196

emmaine@syr.edu

DOI: 10.1534/g3.115.019828

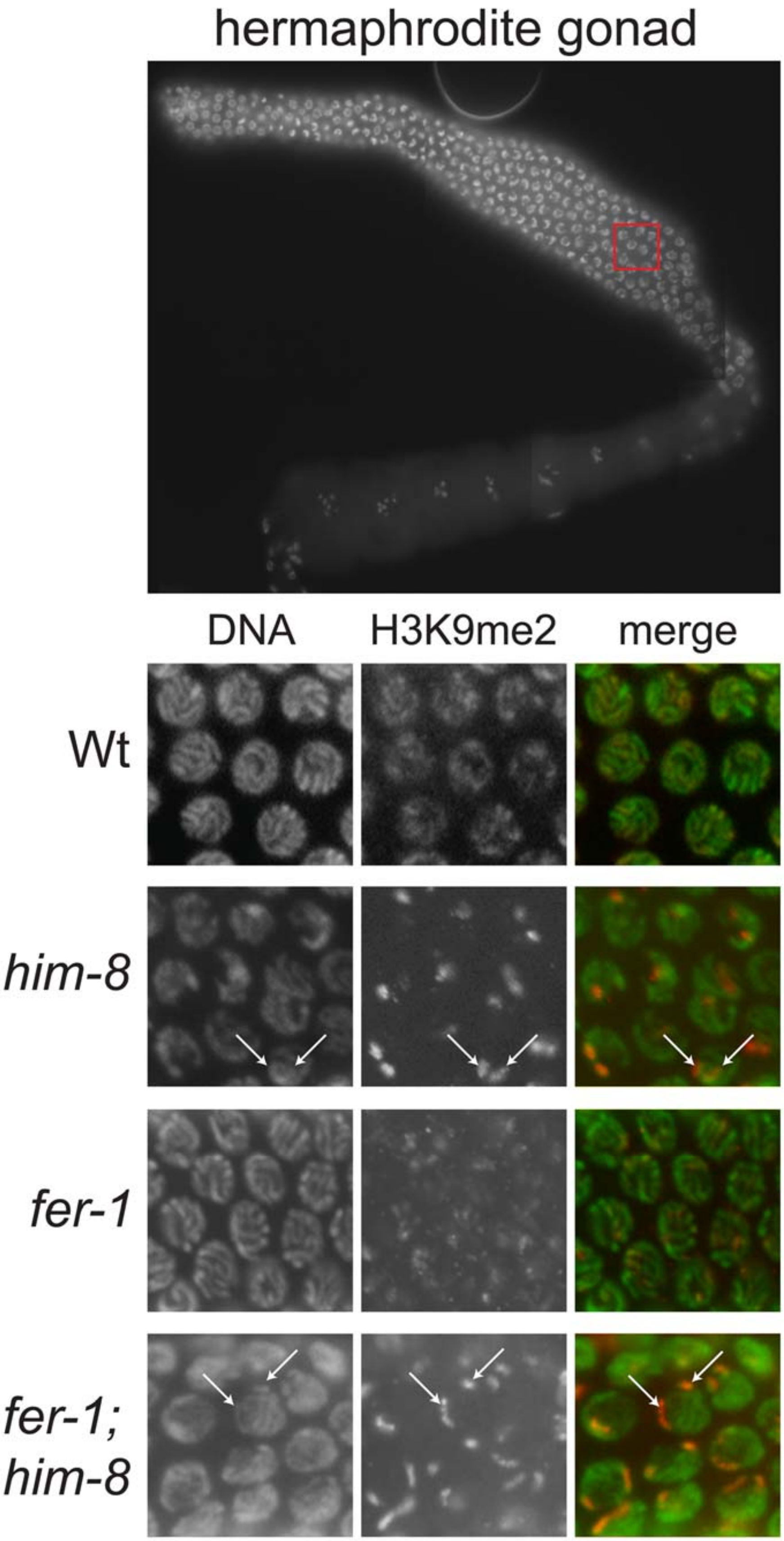
hermaphrodite gonad
DNA
H3K9me2
merge
Wt
him-8
fer-1
fer-1; him-8

Figure S1 H3K9me2 is enriched on the unsynapsed X chromosomes in *fer-1;him-8* mutants. Upper panel, dissected adult hermaphrodite gonad arm stained with DAPI to visualize DNA. Lower panels show pachytene germ cells, from a region corresponding to the boxed region above, co-labeled to detect DNA and H3K9me2. Stronger H3K9me2 foci are visible in *him-8* and *fer-1;him-8* nuclei, corresponding to the X chromosomes.

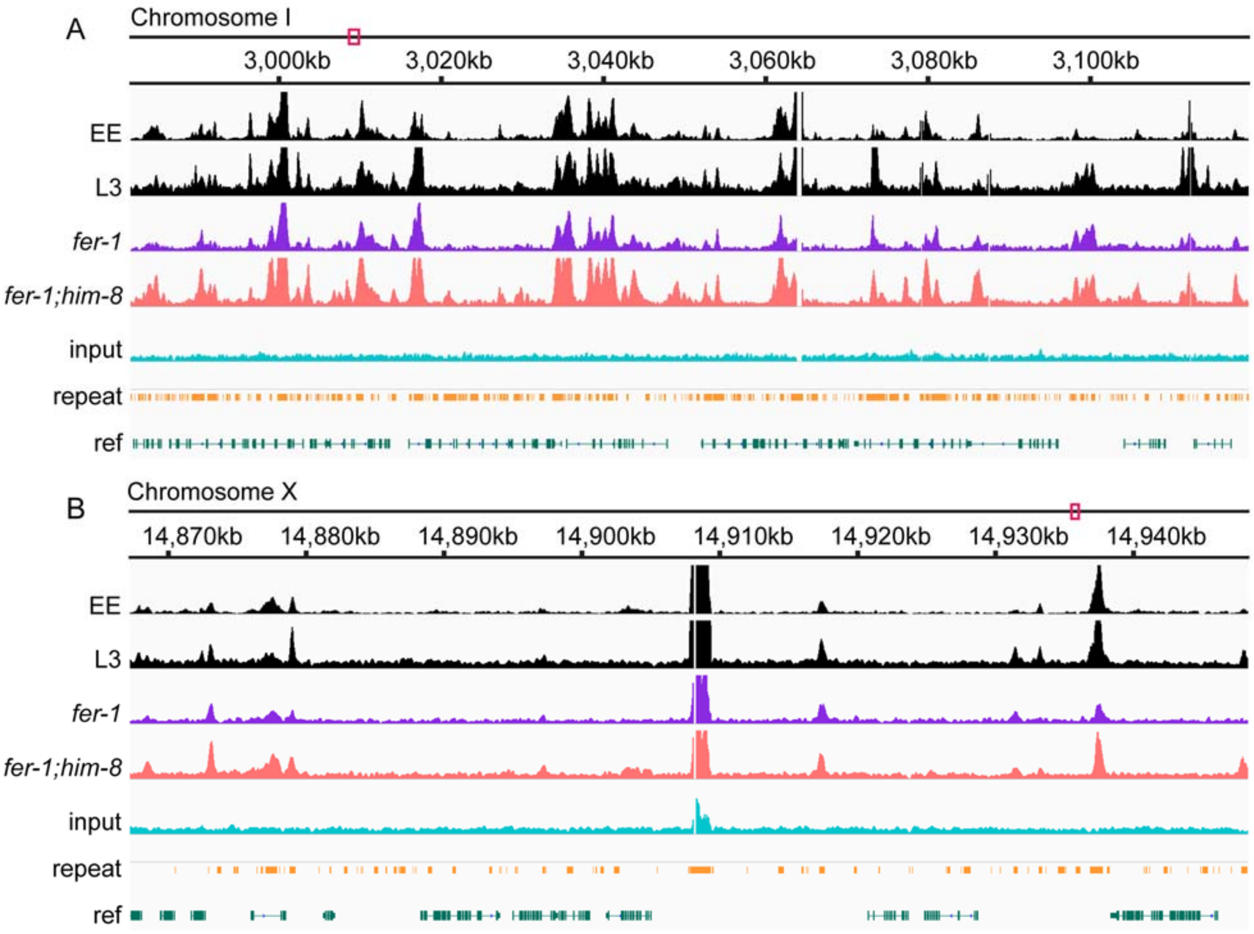

Figure S2 Comparison of H3K9me2 distribution in our adult datasets with L3 larval and early embryo datasets from modENCODE. Screenshots from the Genome Browser show H3K9me2 signal at representative regions of the genome. Peaks tend to correlate with repetitive sequences, although many repeat regions lack H3K9me2. Y-axis scale reflects the number of reads, ranging from a minimum of 0 to a maximum of ≥ 120.

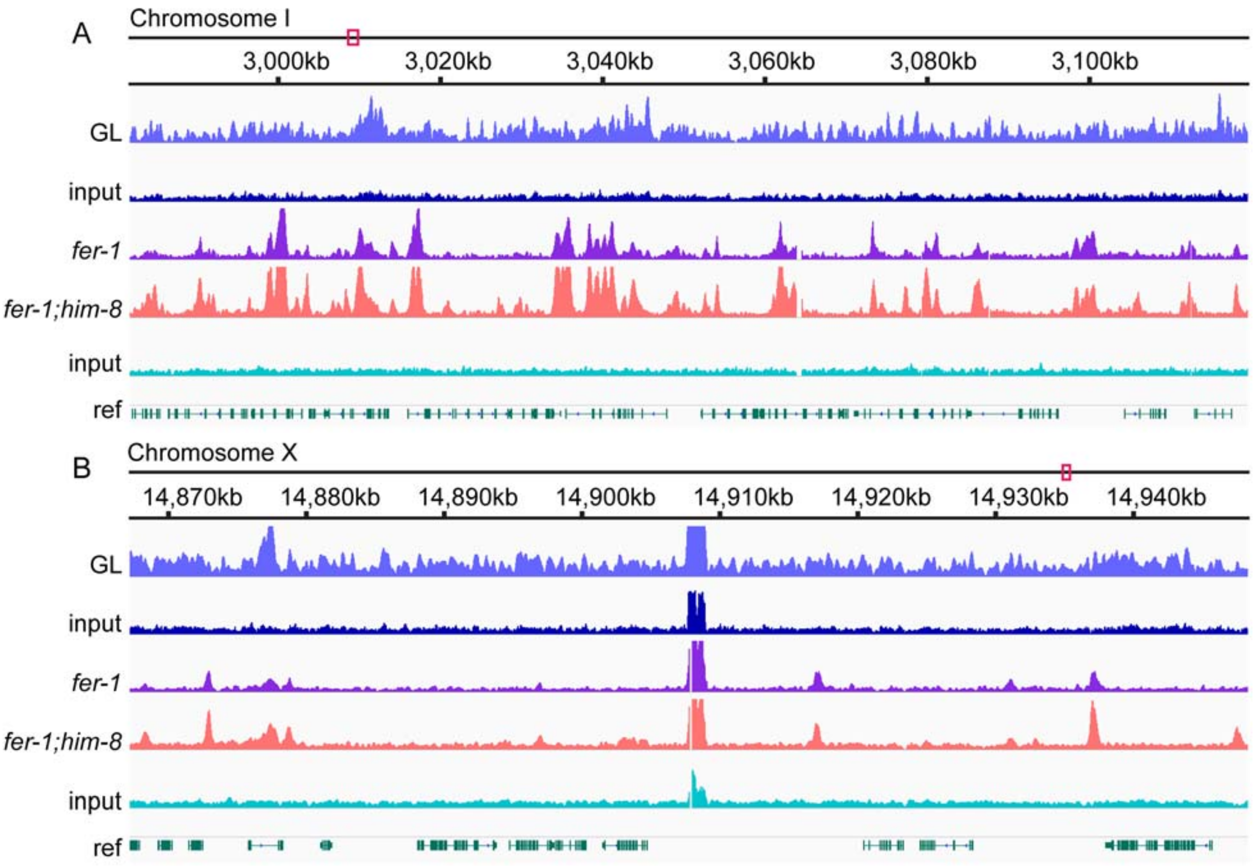

Figure S3 Comparison of H3K9me2 distribution in our adult whole animal datasets with isolated adult germ cell dataset from modENCODE. Screenshots form the Genome Browser show H3K9me2 signal at the same representative regions of the genome as shown in Figure S2. Y-axis scale reflects the number of reads, ranging from a minimum of 0 to a maximum of ≥ 120. GL, germ cell data.

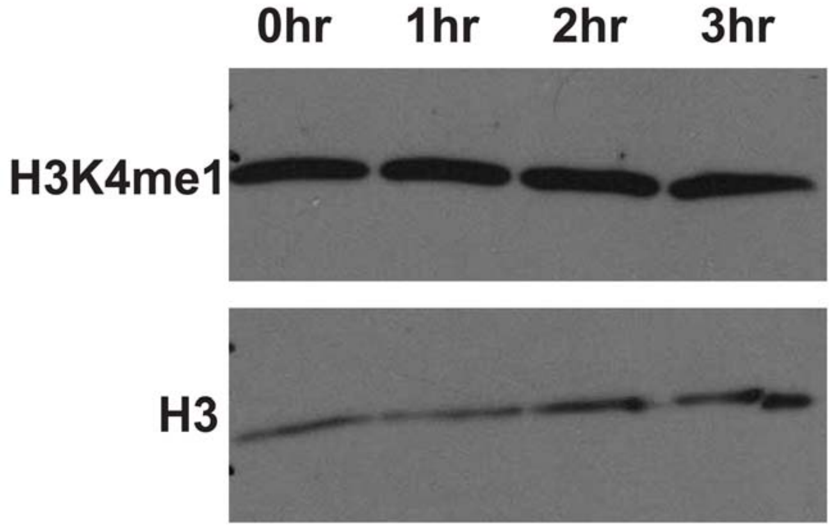

Figure S4 Histone signal does not degrade in dissected tissue. Protein blot indicating H3K4me1 and pan-H3 levels in dissected tissue. Adults were dissected and tissue was allowed to sit in buffer on ice for 0-3 hr, as indicated. An equal number of animals was dissected for each time point, and an equivalent proportion of each sample was loaded onto one gel for pan-H3 detection and another gel for H3K4me1 detection.

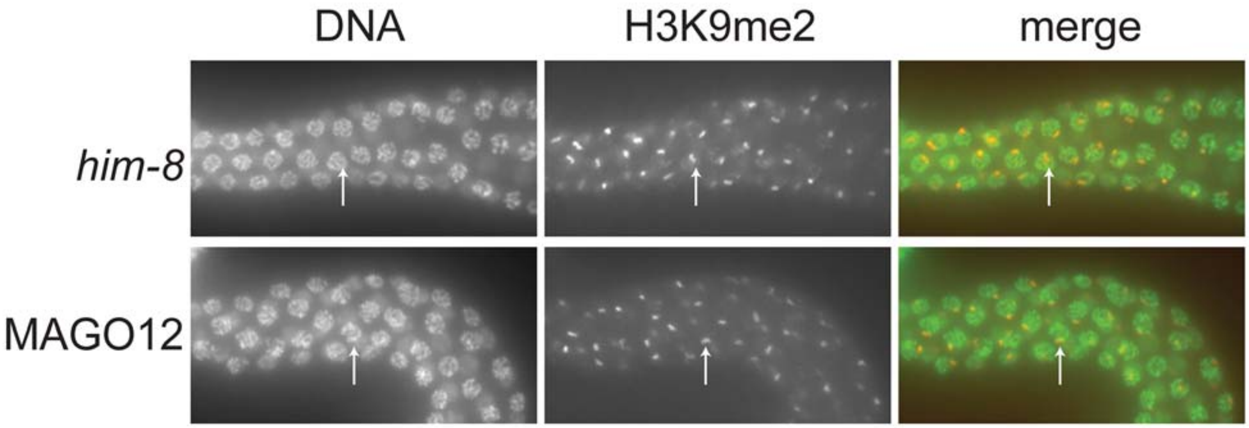

Figure S5 H3K9me2 is enriched on male the X chromosome in MAGO12 mutants during first meiotic prophase. Male gonads were dissected, and H3K9me2 was visualized via indirect immunofluorescence. DNA was visualized with DAPI. Panels show a portion of the late pachytene/early diplotene germline. A single strong focus of H3K9me2 visible in each control and MAGO-12 nucleus (arrows) corresponds to the X chromosome. H3K9me2 signal decreases as nuclei move into diplotene.

Table S1 H3K9me2 tends to be enriched at repetitive DNA sequences

	% total genome	% H3K9me2-enriched genome *fer-1; him-8*	*him-8*
genome	100.00	100.00	100.00
Total repeated sequences^	19.6	53.3	56.8
Retro-elements*	0.89	2.89	2.85
Transposons*	7.10	22.37	22.93
Helitrons*	1.40	5.36	5.83
Unclassified interspersed repeats*	1.24	3.16	3.48
Satellite sequences*	1.03	2.32	2.94
Simple repeats*	1.19	1.71	1.77
Low complexity repeats*	0.30	0.23	0.19
Other repeated sequence**	6.45	15.26	16.81

^ Data obtained from www.wormbase.org. Repeat sequences were identified by Repeat Masker (repeatmasker.org) analysis of genome data and are listed in wormbase as inverted repeats, tandem repeats, or "repeat regions." * Repeat sequences identified by analyzing the genome sequence and H3K9me2 ChIP-enriched sequences for annotated repeats listed in Repbase (www.girinst.org/repbase/ version 20140131) using Repeat Masker. ** Repeat sequences identified by Repeat Masker analysis of the genome sequence (included in "total repeated sequences"), but not annotated in Repbase. See Methods.

Table S2 Developmental defects observed in *wago-1*, *met-2*, and *wago-1;met-2* XX adults

Phenotype	% mutant (n)*		
	wago-1	***met-2***	***wago-1;met-2***
Endomitotic oocytes	3 (69)	9 (90)	5 (56)
Clumped meiotic nuclei	0 (69)	1 (90)	4 (56)
Abnormal meiotic morphology	1 (69)	2 (90)	4 (56)
Defective egg-laying	1 (78)	5 (84)	9 (85)

* n=number of gonad arms for germline defects; n=number of animals for egg-laying defect.

Table S3 Oligonucleotide primers used for real-time PCR reactions.

Name	Sequence	Chromosome	Used with
**P78**	TTCCTCGGGAGATTTTAGCC	X:196839..196858	P79
**P79**	ATCCGGTGTTTAGGGGTACTG	X:196963..196943	
**P80**	CCGAATTCTAGGTAACGGACTG	X:7942473..7942494	P81
**P81**	TCAAGTCTGAGAAGAGCTGGTG	X:7942595..7942574	
**P82**	AATGGTTGCTCTCTTTCTCAGC	X:15922939..15922960	P83
**P83**	GGCCAGTAACCAGAAAGAGTTG	X:15923092..15923071	
**P84**	TTTCCGCCACTCATAGTTGTC	X:15920286..15920306	P85
**P85**	AATTCACTGGCTGGATGGTC	X:15920426..15920407	
