# Supplementary material for: Enrichment of H3K9me2 on Unsynapsed Chromatin in Caenorhabditis elegans Does Not Target de Novo Sites
Source: G3 (Bethesda). 2015 Jul 8;5(9):1865–78. doi: 10.1534/g3.115.019828 (PMC4555223; doi:10.1534/g3.115.019828)
Supplement: Supporting Information [file supp_g3.115.019828_FigureS1.pdf]

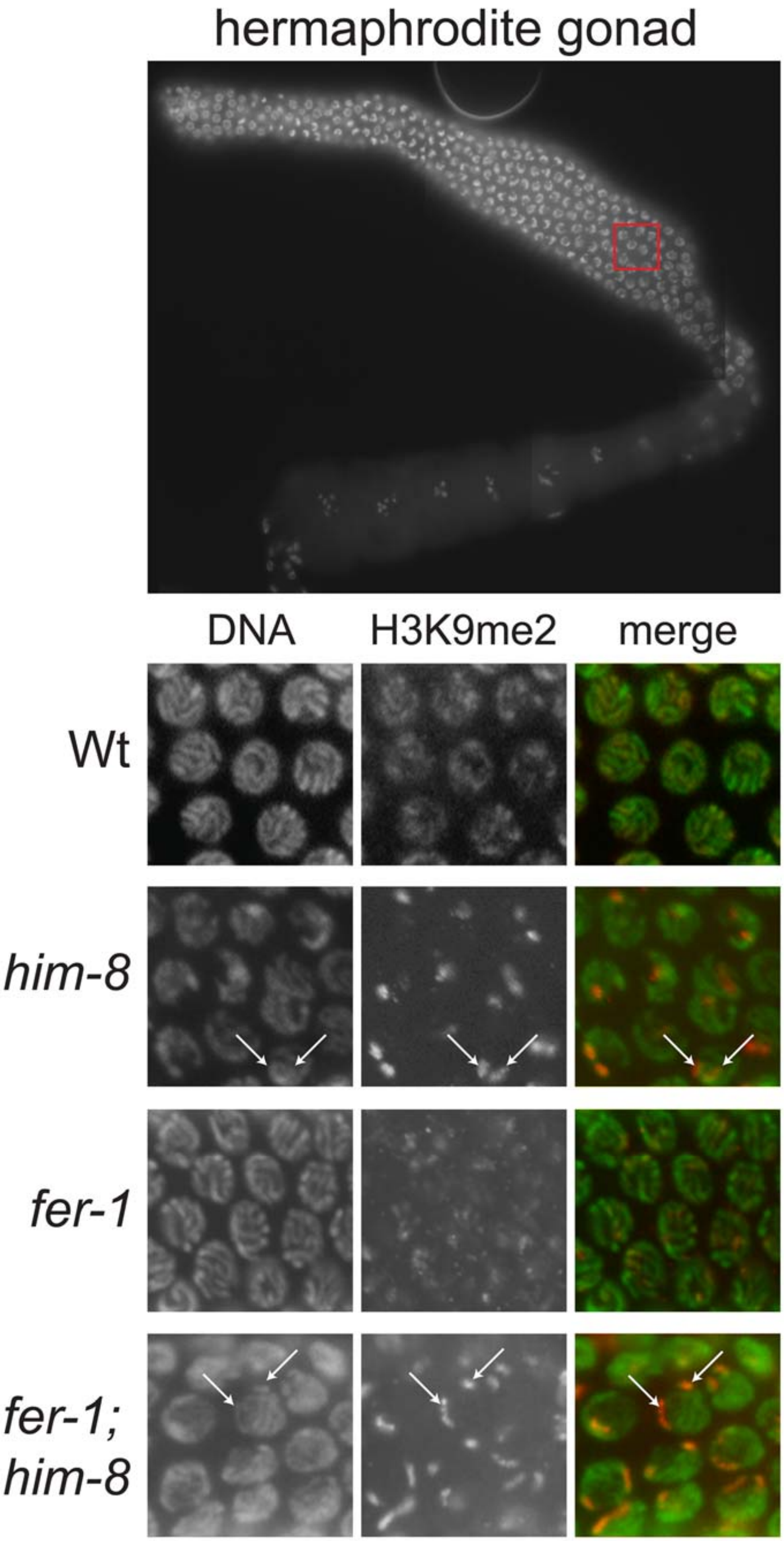
hermaphrodite gonad
DNA
H3K9me2
merge
Wt
him-8
fer-1
fer-1;
him-8

Figure S1 H3K9me2 is enriched on the unsynapsed X chromosomes in *fer-1;him-8* mutants. Upper panel, dissected adult hermaphrodite gonad arm stained with DAPI to visualize DNA. Lower panels show pachytene germ cells, from a region corresponding to the boxed region above, co-labeled to detect DNA and H3K9me2. Stronger H3K9me2 foci are visible in *him-8* and *fer-1;him-8* nuclei, corresponding to the X chromosomes.
