# Supplementary material for: Enrichment of H3K9me2 on Unsynapsed Chromatin in Caenorhabditis elegans Does Not Target de Novo Sites
Source: G3 (Bethesda). 2015 Jul 8;5(9):1865–78. doi: 10.1534/g3.115.019828 (PMC4555223; doi:10.1534/g3.115.019828)
Supplement: Supporting Information [file supp_g3.115.019828_FigureS2.pdf]

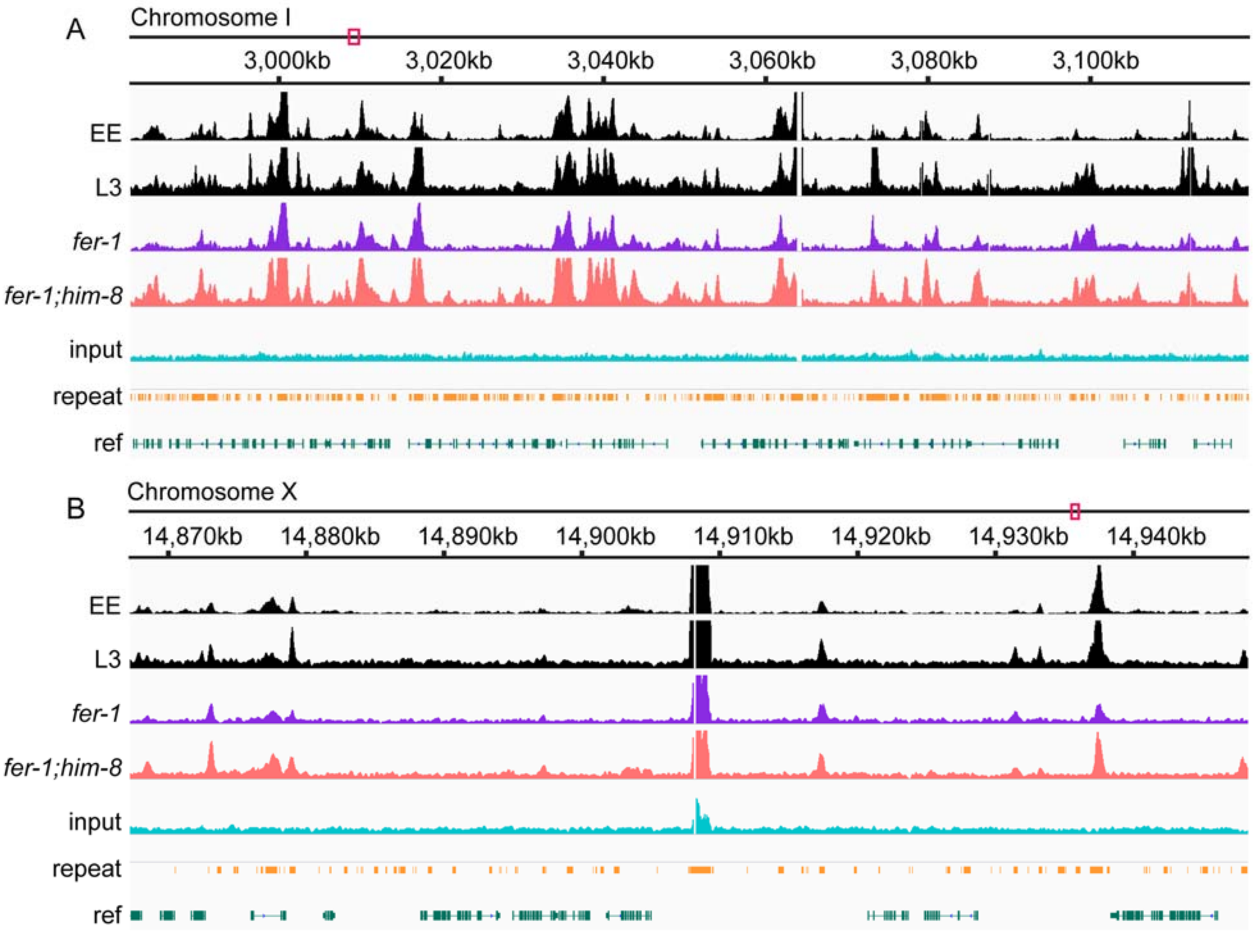

Figure S2 Comparison of H3K9me2 distribution in our adult datasets with L3 larval and early embryo datasets from modENCODE. Screenshots from the Genome Browser show H3K9me2 signal at representative regions of the genome. Peaks tend to correlate with repetitive sequences, although many repeat regions lack H3K9me2. Y-axis scale reflects the number of reads, ranging from a minimum of 0 to a maximum of ≥ 120.
