# Supplementary material for: Enrichment of H3K9me2 on Unsynapsed Chromatin in Caenorhabditis elegans Does Not Target de Novo Sites
Source: G3 (Bethesda). 2015 Jul 8;5(9):1865–78. doi: 10.1534/g3.115.019828 (PMC4555223; doi:10.1534/g3.115.019828)
Supplement: Supporting Information [file supp_g3.115.019828_FigureS3.pdf]

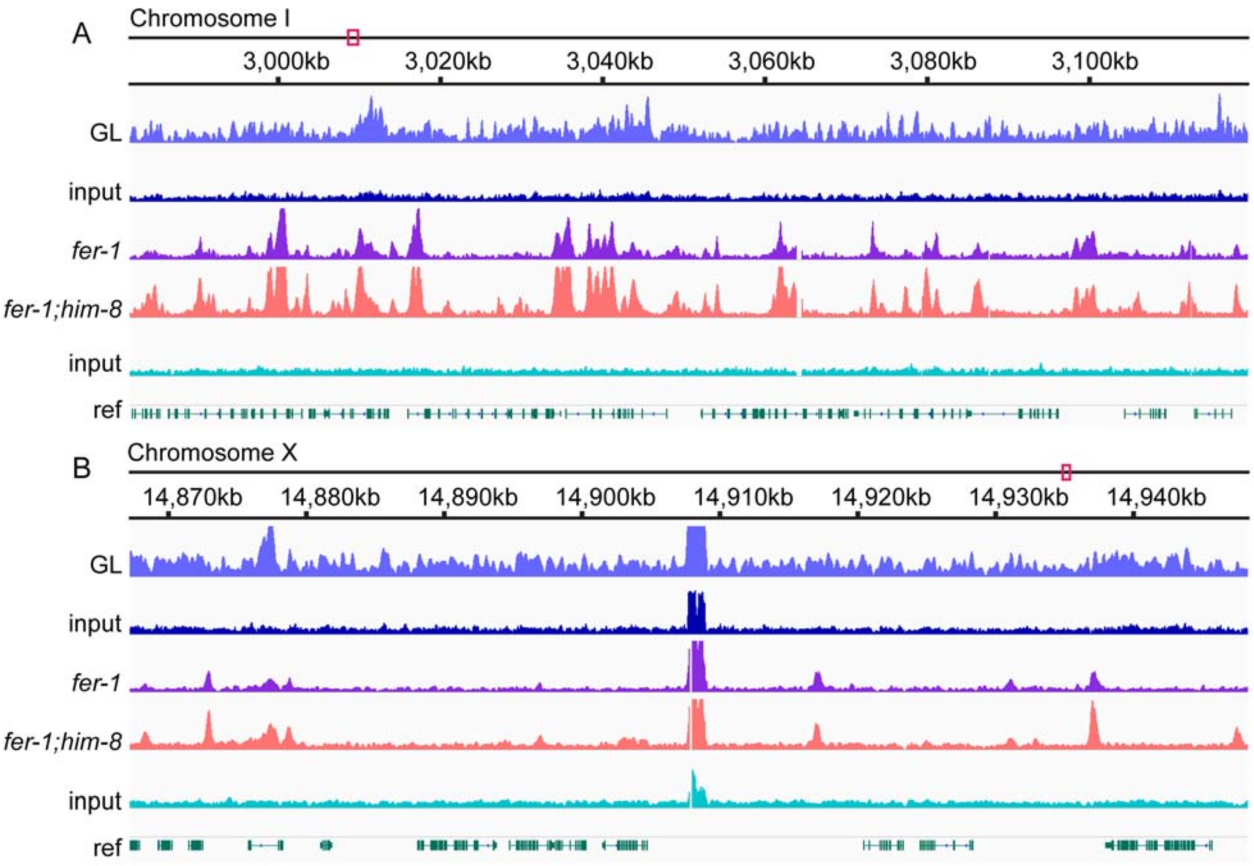

Figure S3 Comparison of H3K9me2 distribution in our adult whole animal datasets with isolated adult germ cell dataset from modENCODE. Screenshots form the Genome Browser show H3K9me2 signal at the same representative regions of the genome as shown in Figure S2. Y-axis scale reflects the number of reads, ranging from a minimum of 0 to a maximum of $\geq$ 120. GL, germ cell data.
