# Supplementary material for: Enrichment of H3K9me2 on Unsynapsed Chromatin in Caenorhabditis elegans Does Not Target de Novo Sites
Source: G3 (Bethesda). 2015 Jul 8;5(9):1865–78. doi: 10.1534/g3.115.019828 (PMC4555223; doi:10.1534/g3.115.019828)
Supplement: Supporting Information [file supp_g3.115.019828_FigureS4.pdf]

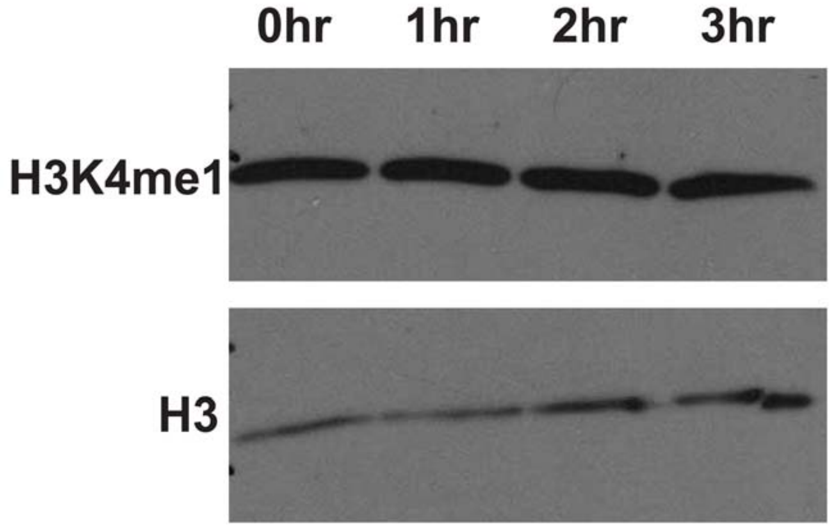

Figure S4 Histone signal does not degrade in dissected tissue. Protein blot indicating H3K4me1 and pan-H3 levels in dissected tissue. Adults were dissected and tissue was allowed to sit in buffer on ice for 0-3 hr, as indicated. An equal number of animals was dissected for each time point, and an equivalent proportion of each sample was loaded onto one gel for pan-H3 detection and another gel for H3K4me1 detection.
