# Supplementary material for: Enrichment of H3K9me2 on Unsynapsed Chromatin in Caenorhabditis elegans Does Not Target de Novo Sites
Source: G3 (Bethesda). 2015 Jul 8;5(9):1865–78. doi: 10.1534/g3.115.019828 (PMC4555223; doi:10.1534/g3.115.019828)
Supplement: Supporting Information [file supp_g3.115.019828_FigureS5.pdf]

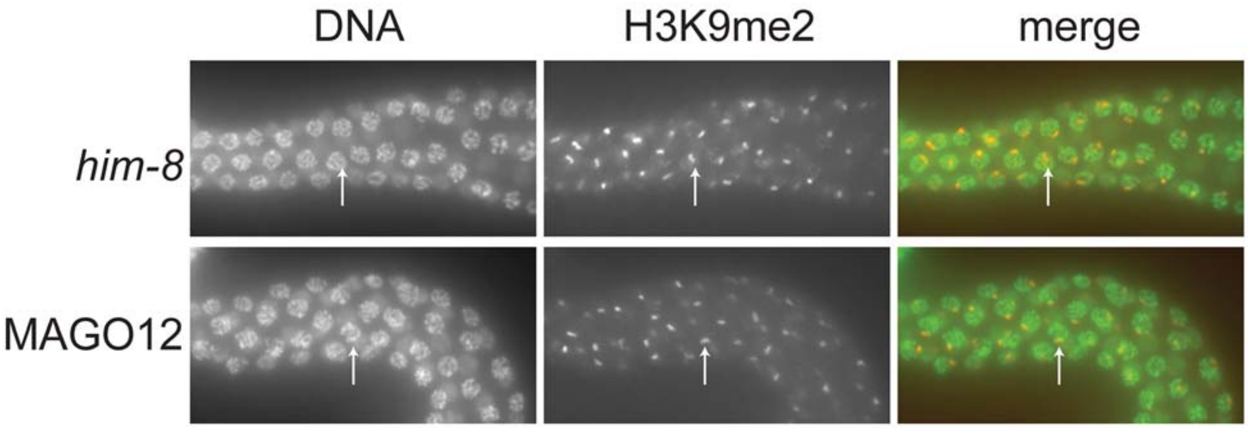

Figure S5 H3K9me2 is enriched on male the X chromosome in MAGO12 mutants during first meiotic prophase. Male gonads were dissected, and H3K9me2 was visualized via indirect immunofluorescence. DNA was visualized with DAPI. Panels show a portion of the late pachytene/early diplotene germline. A single strong focus of H3K9me2 visible in each control and MAGO-12 nucleus (arrows) corresponds to the X chromosome. H3K9me2 signal decreases as nuclei move into diplotene.
