# Supplementary material for: Enrichment of H3K9me2 on Unsynapsed Chromatin in Caenorhabditis elegans Does Not Target de Novo Sites
Source: G3 (Bethesda). 2015 Jul 8;5(9):1865–78. doi: 10.1534/g3.115.019828 (PMC4555223; doi:10.1534/g3.115.019828)
Supplement: Supporting Information [file supp_g3.115.019828_TableS1.pdf]

Table S1 H3K9me2 tends to be enriched at repetitive DNA sequences

	% total genome	% H3K9me2-enriched genome *fer-1; him-8*	*him-8*
genome	100.00	100.00	100.00
Total repeated sequences^	19.6	53.3	56.8
Retro-elements*	0.89	2.89	2.85
Transposons*	7.10	22.37	22.93
Helitrons*	1.40	5.36	5.83
Unclassified interspersed repeats*	1.24	3.16	3.48
Satellite sequences*	1.03	2.32	2.94
Simple repeats*	1.19	1.71	1.77
Low complexity repeats*	0.30	0.23	0.19
Other repeated sequence**	6.45	15.26	16.81

^ Data obtained from www.wormbase.org. Repeat sequences were identified by Repeat Masker (repeatmasker.org) analysis of genome data and are listed in wormbase as inverted repeats, tandem repeats, or "repeat regions." * Repeat sequences identified by analyzing the genome sequence and H3K9me2 ChIP-enriched sequences for annotated repeats listed in Repbase (www.girinst.org/repbase/ version 20140131) using Repeat Masker. ** Repeat sequences identified by Repeat Masker analysis of the genome sequence (included in "total repeated sequences"), but not annotated in Repbase. See Methods.
