# Supplementary material for: Enrichment of H3K9me2 on Unsynapsed Chromatin in Caenorhabditis elegans Does Not Target de Novo Sites
Source: G3 (Bethesda). 2015 Jul 8;5(9):1865–78. doi: 10.1534/g3.115.019828 (PMC4555223; doi:10.1534/g3.115.019828)
Supplement: Supporting Information [file supp_g3.115.019828_TableS2.pdf]

Table S2 Developmental defects observed in *wago-1*, *met-2*, and *wago-1;met-2* XX adults

**Phenotype**	**% mutant (n)***		
	wago-1	***met-2***	***wago-1;met-2***
Endomitotic oocytes	3 (69)	9 (90)	5 (56)
Clumped meiotic nuclei	0 (69)	1 (90)	4 (56)
Abnormal meiotic morphology	1 (69)	2 (90)	4 (56)
Defective egg-laying	1 (78)	5 (84)	9 (85)

* n=number of gonad arms for germline defects; n=number of animals for egg-laying defect.
