# Supplementary material for: Enrichment of H3K9me2 on Unsynapsed Chromatin in Caenorhabditis elegans Does Not Target de Novo Sites
Source: G3 (Bethesda). 2015 Jul 8;5(9):1865–78. doi: 10.1534/g3.115.019828 (PMC4555223; doi:10.1534/g3.115.019828)
Supplement: Supporting Information [file supp_g3.115.019828_TableS3.pdf]

Table S3 Oligonucleotide primers used for real-time PCR reactions.

Name	Sequence	Chromosome	Used with
**P78**	TTCCTCGGGAGATTTTAGCC	X:196839..196858	P79
**P79**	ATCCGGTGTTTAGGGGTACTG	X:196963..196943	
**P80**	CCGAATTCTAGGTAACGGACTG	X:7942473..7942494	P81
**P81**	TCAAGTCTGAGAAGAGCTGGTG	X:7942595..7942574	
**P82**	AATGGTTGCTCTCTTTCTCAGC	X:15922939..15922960	P83
**P83**	GGCCAGTAACCAGAAAGAGTTG	X:15923092..15923071	
**P84**	TTTCCGCCACTCATAGTTGTC	X:15920286..15920306	P85
**P85**	AATTCACTGGCTGGATGGTC	X:15920426..15920407	
